# Toward a Bidirectional Communication Between Retinal Cells and a Prosthetic Device – A Proof of Concept

**DOI:** 10.3389/fnins.2019.00367

**Published:** 2019-04-30

**Authors:** Viviana Rincón Montes, Jana Gehlen, Stefan Lück, Wilfried Mokwa, Frank Müller, Peter Walter, Andreas Offenhäusser

**Affiliations:** ^1^Bioelectronics, Institute of Complex Systems-8, Forschungszentrum Jülich, Jülich, Germany; ^2^Cellular Biophysics, Institute of Complex Systems-4, Forschungszentrum Jülich, Jülich, Germany; ^3^Department of Materials in Electrical Engineering 1, RWTH Aachen University, Aachen, Germany; ^4^Department of Ophthalmology, RWTH Aachen University, Aachen, Germany

**Keywords:** retinal implants, intraretinal implants, penetrating microelectrode array, retinal recordings, retinal stimulation, bidirectional communication

## Abstract

**Background:** Significant progress toward the recovery of useful vision in blind patients with severe degenerative retinal diseases caused by photoreceptor death has been achieved with the development of visual prostheses that stimulate the retina electrically. However, currently used prostheses do not provide feedback about the retinal activity before and upon stimulation and do not adjust to changes during the remodeling processes in the retina. Both features are desirable to improve the efficiency of the electrical stimulation (ES) therapy offered by these devices. Accordingly, devices that not only enable ES but at the same time provide information about the retinal activity are beneficial. Given the above, a bidirectional communication strategy, in which inner retinal cells are stimulated and the output neurons of the retina, the ganglion cells, are recorded using penetrating microelectrode arrays (MEAs) is proposed.

**Methods:** Custom-made penetrating MEAs with four silicon-based shanks, each one with three or four iridium oxide electrodes specifically designed to match retinal dimensions were used to record the activity of light-adapted wildtype mice retinas and degenerated retinas from *rd10* mice *in vitro*. In addition, responses to high potassium concentration and to light stimulation in wildtype retinas were examined. Furthermore, voltage-controlled ES was performed.

**Results:** The spiking activity of retinal ganglion cells (RGCs) was recorded at different depths of penetration inside the retina. Physiological responses during an increase of the extracellular potassium concentration and phasic and tonic responses during light stimulation were captured. Moreover, pathologic rhythmic activity was recorded from degenerated retinas. Finally, ES of the inner retina and simultaneous recording of the activity of RGCs was accomplished.

**Conclusion:** The access to different layers of the retina with penetrating electrodes while recording at the same time the spiking activity of RGCs broadens the use and the field of action of multi-shank and multi-site penetrating MEAs for retinal applications. It enables a bidirectional strategy to stimulate inner retinal cells electrically and to record from the spiking RGCs simultaneously (BiMEA). This opens the possibility of a feedback loop system to acknowledge the success of ES carried out by retinal prostheses.

## Introduction

The retina harbors not only the photoreceptors but also a neuronal network (“inner retina”) that serves for information processing and provides the retinal output neurons, the retinal ganglion cells (RGCs). Degenerative retinal diseases caused by photoreceptor death, such as age-related macular degeneration (AMD) and retinitis pigmentosa (RP), are the third leading cause of blindness in the world (Hartong et al., 2006; Pascolini and Mariotti, 2012). Nowadays it is not possible to restore full vision, yet multiple efforts have been made to treat blind patients with photoreceptors loss. Therapeutic and experimental strategies range from vitamins and pharmacotherapies to transplantation of lost retinal tissue, stem-cell based therapies, gene-replacement, and visual prostheses (Hartong et al., 2006; Mills et al., 2017). The latter consist of devices that perform electrical stimulation (ES) to different locations of the visual pathway, including mainly the visual cortex, the optic nerve, and the retina (Margalit et al., 2002; Lewis et al., 2015). In cases in which photoreceptors are lost but the remaining inner retina is still intact, retinal implants have been used with significant advancements toward the restoration of useful vision in blind patients (Zrenner, 2013; Cheng et al., 2017).

In order to restore the lost function of photoreceptors, commercially available retinal prostheses comprise primarily of a light-sensing device and a microelectrode array (MEA). To capture visual information, a camera together with a signal processor unit can be used to detect and process light stimuli. Likewise, a light-sensitive device such as an array of photodiodes might be used. Following, visual information is transduced into electrical signals and conducted into the retinal tissue by a pulse generator or induced by the same array of photodiodes through a MEA. Thereby, retinal implants activate the remaining circuitry of the visual pathway due to the ES of bipolar and/or RGCs, depending on whether the corresponding MEA is placed between the sclera and the choroid (suprachoroidal), between the choroid and the remaining retinal cells (subretinal), or between the neural layer of the retina and the vitreous body (epiretinal) (Margalit et al., 2002; Zrenner, 2013).

While current retinal prostheses allow the restoration of useful visual percepts to blind patients with RP, they do not automatically adjust to the remodeling processes of the retina that lead to an increase of the stimulation threshold and a reduction of the efficiency of the ES therapy of these devices (Cheng et al., 2017; Haselier et al., 2017). Furthermore, it is not clear how visual signals are being encoded in the visual pathway during stimulation, what becomes even more difficult when retinal areas are being stimulated with large surface area electrodes that allow the stimulation of multiple retinal cells after one stimulation pulse (Cheng et al., 2017). Accordingly, devices that not only enable ES but at the same time provide feedback of the retinal activity are beneficial.

With the aim to facilitate the improvement and adjustment of ES parameters in retinal implants and to provide means to examine in real-time the electrical activity within the retina, a bidirectional communication strategy between retinal cells and a prosthetic device using a penetrating MEA has been proposed (Heil et al., 2014; Brusius, 2015; Walter, 2016). In this way, the possibility for simultaneous recording and stimulation is opened.

**FIGURE 1:**
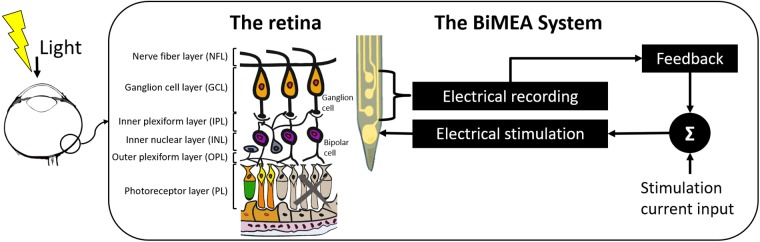
Concept of the BiMEA system. The schematic shows a bidirectional microelectrode array (BiMEA), which consists of a multi-site penetrating MEA, allowing the system to perform electrical stimulation to the inner retina (from the inner plexiform layer to the outer margin of the inner nuclear layer) and to record the electrical activity of retinal ganglion cells (RGCs).

In this work, a proof of concept confirming the feasibility of using penetrating MEAs as a dual purpose device that stimulates the inner retina and records local field potentials and spiking activity of retinal ganglion cells (RGCs) is unveiled (see Figure 1). Upon photoreceptor degeneration, the thickness of the retina is reduced to approximately 100 μm. Therefore, penetrating devices have to be specifically tailored to match these dimensions. The work presented here shows the design of the probe, its fabrication principles and the application *in vitro* in retinas of normal mice and mice showing a retinal degeneration. Furthermore, the potential field of action of penetrating MEAs in retinal applications is shown. As they allow access to the different retinal layers, a follow-up of a group of neurons corresponding to a same neuronal column is possible.

## Materials and Methods

### The BiMEA Probes

Custom-made penetrating MEAs, here after called BiMEAs, were designed and fabricated at the *Institut für Werkstoffe der Elektrotechnik (IWE-1)*, RWTH Aachen University (Germany) after evaluating the design by Brusius (2015) fabricated by NeuroNexus (Michigan, United States).

#### Design and Fabrication

**FIGURE 2:**
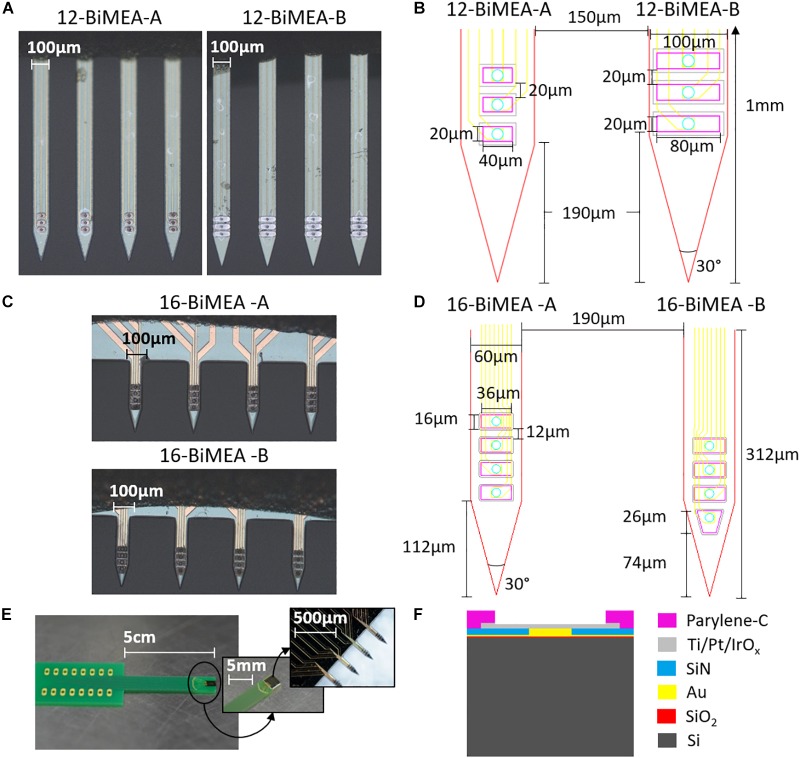
The BiMEA probes. Optical images of the BiMEA probes **(A,C)** bonded to a carrier with a 16-DIP connector **(E)** are shown. The dimensions for both 12-BiMEA shank designs are depicted in **(B)**, which have a distance of 190 μm from the tip to the first electrode (DTE). In contrast, the 16-BiMEAs are displayed in **(D)**, showing a 16-BiMEA-A shank at the left and a 16-BiMEA-B shank at the right with DTEs of 112 μm and 74 μm, respectively. Additionally, the cross-section of a single shank is depicted in **F**.

The BiMEA probes are penetrating MEAs with four silicon-based shanks, each one containing three or four iridium oxide (IrO_x_) electrodes. In total, each penetrating structure contains 12 or 16 electrodes, named 12-BiMEA or 16-BiMEA, respectively. The 12-BiMEAs belong to the first generation of such probes, which have 10–20 μm thick shanks with a width of 100 μm, an inter-shank distance of 150 μm, a total shank length of 1 mm, and rectangular electrodes with a surface area of either 800 μm^2^ (12-BiMEA-A) or 1600 μm^2^ (12-BiMEA-B) and a vertical inter-electrode distance of 20 μm (Figure 2A,B). The 16-BiMEAs have narrower shanks with a width of 60 μm, a total length of 312 μm, and an inter-shank distance of 190 μm. In contrast to the first design, the 16-BiMEAs have either four rectangular electrodes with a surface area of 576 μm^2^ (16-BiMEA-A) or three rectangular electrodes and a bottom trapezoid electrode with the same surface area (16-BiMEA-B) (Figure 2C,D). In order to facilitate the insertion of the shanks, both BiMEA designs have a tip angle of 30°. Each silicon (Si) structure is additionally bonded and glued to a carrier with a 16-DIP connector (Figure 2E).

Because of the small area of the stimulation sites, the electrode material must fulfill certain features. The necessary charge must be delivered to evoke action potentials therefore a high charge delivery capacity is needed. At the same time the voltage range has to be kept in a safe range to prevent irreversible electrode alterations and electrolysis in the interstitial fluid. Therefore, IrO_x_ was chosen as the electrode material, as it fulfills all of the aforementioned requirements (Slavcheva et al., 2004, 2006; Wessling et al., 2006; Van Ooyen et al., 2009).

Each Si shank is made of a 20 μm Si substrate, followed by a 300 nm thick layer of oxidized silicon (SiO_2_), Titanium/Gold (Ti/Au) feedlines 30/300 nm thick, a first passivation layer of silicon nitride (SiN) 1 μm thick with an opening filled with Au. On top, a stack layer with 30 nm of Ti, 250 nm of platinum (Pt), and 500 nm of IrO_x_ form the surface area of the electrodes. Afterward, a coating of 3 μm of parylene-C forms a second passivation layer with the corresponding openings to expose the surface area of the IrOx electrodes (Figure 2F). For the purpose of this work, the four types of BiMEA probes were used indistinctively during the experiments, however, for direct comparisons between experiments the 16-BiMEAs were used.

To keep track of the recordings corresponding to a same vertical column, the shanks of the BiMEA probes were numbered from 1 to 4 from right to left, and the electrodes were numbered from 1 to 4 from the bottom to the top. Thus, while electrode 1.1 (E_1.1_) corresponds to the bottom electrode of the right-most shank and E_1.4_ to the top electrode, E_4.4_ is the top electrode of the left-most shank.

#### Electrochemical Properties

The IrO_x_ electrodes were electrochemically activated using an EG&G 283 Potentiostat/Galvanostat (AMETEK Scientific Instruments) via cyclic voltammetry (CV) with 500 cycles, a scan rate of 100 mV/s, and activation potentials from -0.85 to 0.85 V versus a Silver/Silver Chloride (Ag/AgCl) reference electrode in 0.9% phosphate buffered saline solution (PBS). In addition, the charge delivery capacity (Q_dc_) was calculated at the last cycle of the CV curve integrating the current density along the electrode potential versus the Ag/AgCl electrode as suggested by Slavcheva et al. (2004). The BiMEA probes showed a Q_dc_ between 239.3 and 552.5 mC/cm^2^.

The impedance of the electrodes was evaluated by electrochemical impedance spectroscopy (EIS) using a potentiostat (VSP-300, Bio-Logic Science Instruments SAS) and a three-electrode cell setup prior to the first usage. Each IrOx electrode served as a working electrode and a Ag/AgCl electrode and a platinum (Pt) wire were used as reference and counter electrodes, respectively. The EIS measurements were carried out in 10xPBS applying a 10 mV sinus wave in a range of frequencies between 1 Hz and 100 kHz. The IrO_x_ electrodes showed a low impedance, which decreased with respect to the increasing electrode surface area (ESA) of the BiMEA electrodes, especially in the frequency range of interest where neuronal spikes are captured (10^2^–10^3^ Hz). Electrochemical properties showed by the BiMEA probes are summarized in Table 1, and the respective impedance and CV plots are shown in Supplementary Figure 1.

**Table 1:** Summary of the electrochemical properties of the BiMEA probes.

BiMEA probes	ESA [μm^2^]	|Z| @ 1kHz [kΩ]	Q_dc_ [mC/cm^2^]
16-BiMEA	576	33.89 ± 16.09	552.5 ± 9.3
12-BiMEA-A	800	24.24 ± 4.03	243.9 ± 40
12-BiMEA-B	1600	12.26 ± 1.78	239.3 ± 31.5


*ESA stands for electrode surface area, |Z| @ 1 kHz refers to magnitude impedance at 1 kHz, and Q_*dc*_ stands for charge delivery capacity.*

### Animals

Wildtype animals of the strain C57BL/6 were obtained from Charles River and *rd10* mice were bred locally from breeding pairs obtained from Jackson (strain name: B6CXB1-Pde6brd10/J). In this line the *rd10* mutation was backcrossed onto the C57BL/6J background for five generations before intercrossing to homozygosity. All animals were kept on a 12 h light/dark cycle with food and water *ad libitum*. All experiments were performed in accordance with the German Law for the Protection of Animals and after approval was obtained by the regulatory authorities, the Forschungszentrum Jülich and the Landesamt für Natur, Umwelt, und Verbraucherschutz of the German federal state of North-Rhine Westfalia.

### Retina Preparation

Light-adapted retinas from wildtype and *rd10* mice were prepared under ambient light. The animals were deeply anesthetized with isoflurane (Actavis Dtl. GmbH &Co. KG, Germany) and killed by decapitation, followed by the enucleation of the eyeballs, which were immediately transferred into oxygenated Ames’ medium (Sigma-Aldrich, Germany) at room temperature. The physiological solution was bubbled with carbogen gas (The Linde Group, Germany) containing 95% O_2_ and 5% CO_2_ at a pH of 7.4. In order to keep both eyes vital and ensure perfusion during preparation, the eyes were first opened along the *ora serrata*, allowing the removal of the cornea and the lens. Hereafter the procedure that is explained was effected for each eye as the retinal tissue was used for the experiments.

The lens and the vitreous body were carefully removed using fine forceps. Then, the retina was separated from the eyecup and cut into halves. One half was stored in oxygenated Ames’ medium until it was used, and the other piece was mounted with the ganglion cell layer (GCL) facing downwards onto a circular piece of nitrocellulose filter paper (Merck KGaA, Germany). The filter served as a carrier for the tissue and had a precut central hole with a diameter of 1.5 mm. Afterward, the filter/retina sandwich was transferred onto a polydimethylsiloxane (PDMS) pillow with the GCL facing up, and the filter paper was fixed to the PDMS using insect pins. Finally, the tissue was covered with fresh oxygenated medium.

### Experimental Setup

The experimental setup was based on Brusius (2015) and consisted of two main components: a data acquisition system (DAQ) and a measurement chamber.

#### Data Acquisition System

Electrical recordings were performed using the BioMAS (Maybeck et al., 2016), an in-house amplification system with an ES unit that allowed voltage-controlled stimulation of the retina. The system was connected to a 16-channel-headstage that served not only as a pre-amplification stage, but allowed the measurement of the injected current during ES, as it features an internal current measurement circuit that is connected to the respective stimulating electrodes. Moreover, the DAQ provided a digital output for the activation of an LED circuit during light stimulation, and auxiliary channels were used for recording the LED signal, the ES signal, and the current injected to the tissue during ES. Additionally, a high pass filter at 0.1/1 Hz, a sampling rate (F_s_) of either 10 or 20 kHz, and a Ag/AgCl reference electrode were used for the electrical recordings.

#### Measurement Chamber

**FIGURE 3:**
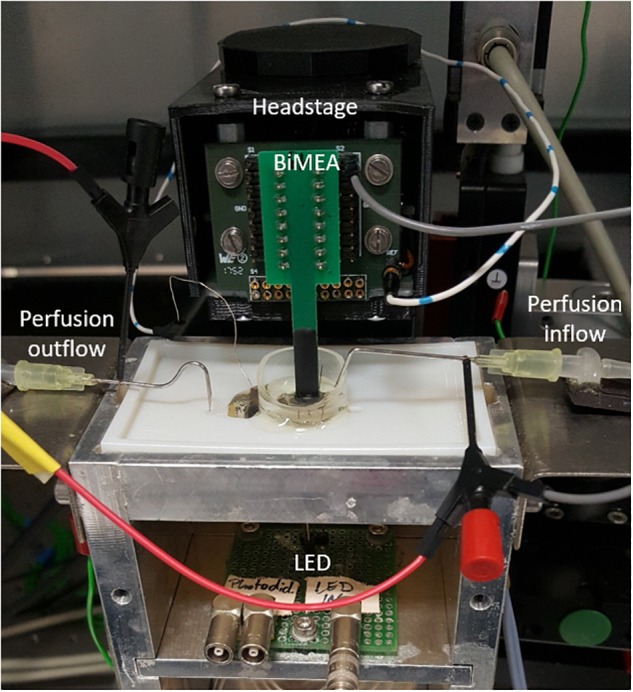
Experimental setup. Inside a Faraday cage, the BioMAS system with a 16-channel-headstage was used for recording the retinal activity and stimulating electrically the retina. The front-end of the headstage and a micromanipulator system were used to place the BiMEA probes inside the tissue. In turn, the retinal tissue was placed inside a perfusion chamber, which had a constant inflow and outflow of oxygenated Ames’ medium. Additionally, a light-emitting diode (LED) circuit was employed to perform light stimulation.

A Faraday cage was used to shield the measurement setup, including a support for holding the perfusion chamber, the headstage of the BioMAS system, whose front-end facilitated the handling of the BiMEAs, a LED circuit, and a micromanipulator system (Luigs & Neumann, Germany), which enabled the movement of the probes along three different axes (x, y, and z). Furthermore, the perfusion chamber comprised a reservoir with a PDMS pillow to support the retinal tissue and allowed the inflow and outflow of oxygenated Ames’ medium at room temperature through a perfusion system with a flow rate between 3 and 4 ml/min, keeping the retina vital during the experiments (see Figure 3).

### *In vitro* Electrophysiology

#### Positioning the BiMEA Inside the Retina

Once the perfusion system was set to run, the BiMEA shanks were driven down slowly with the micromanipulator system onto the epiretinal surface of the tissue until the first peaks or spikes were captured, setting this first position as Z_0_. Then, the insertion was carried out stepwise inside the tissue, at intervals of approximately 20–25 μm. In this way, further depths (Z_x_) inside the tissue were referenced to the electrode that recorded the first electrical activity.

#### Treatment With High Potassium Concentration

The extracellular concentration of potassium (K^+^) was increased during the perfusion of the tissue to initiate depolarization and increase spiking activity of RGCs. To this effect, potassium bicarbonate (KHCO_3_) was added to the regular Ames’s medium solution to achieve a 20 mM K^+^ concentration. The tissue was superfused for 2 min with the high K^+^ solution followed by washout with the regular physiological solution.

#### Light Stimulation

A 500 ms squared pulse with an intensity of 5 V was generated with the BioMAS system to activate the LED circuit, which consisted of a 5 mm round white LED connected in series to a 61.9 Ω resistor. This configuration allowed an LED current of 34.2 mA that produced a power of 7.96 μW/mm^2^ measured at the position of the retina in the recording chamber. This corresponds to a high photopic light stimulus comparable to broad daylight that effectively activates cone photoreceptors. Single and multiple pulses every 15 s were used to perform optical stimulation of the retina.

### Electrical Stimulation

In order to avoid the generation of high voltages that might induce irreversible and undesired reactions like water electrolysis and electrode and tissue damage (Gekeler et al., 2004; Cogan, 2008; Brusius, 2015), a voltage-controlled stimulation was chosen. Moreover, biphasic pulses have been shown to be a good strategy to activate the majority of RGCs, especially in degenerated retinas (Jensen and Rizzo, 2009; Goo et al., 2011b; Celik and Karagoz, 2018). Hence, biphasic squared voltage pulses with an initial cathodic phase followed by an anodic phase were used to carry out ES. Considering the stimulation parameters suggested by different research groups (Walter et al., 2005; Stett et al., 2007; Roessler et al., 2009; Goo et al., 2011b) to perform optimal stimulation of the retina in terms of low charge densities and evoked potentials, pulses with amplitudes of ±600 and ±800 mV and phase durations between 0.5 and 0.8 ms were tested (ES-1: 0.8 mV – 0.5 ms; ES-2: 0.8 mV – 0.6 ms; ES-3: 0.6 mV – 0.5 ms; ES-4: 0.6 mV – 0.6 ms; ES-5: 0.6 mV – 0.7 ms; ES-6: 0.6 mV – 0.8 ms). When performing ES, only one bottom electrode at a time was selected as the stimulating electrode, which was previously positioned in the inner retina. The latter means that the stimulating electrode was barely recording retinal spikes due to its location within the tissue. To test for reproducibility, the tissue was stimulated with six electrical pulses every 20 s.

In order to assess the efficiency of the ES, an electrical stimulation efficiency ratio (ESE) was calculated by dividing the firing rate in a window of 400 ms after the ES artifact by the firing rate averaged in 8 s before the stimulation pulse as proposed by Haselier et al. (2017). ESE values higher than one indicate an increase of the firing rate after ES, while ESE values lower than one show a decrease of the electrical activity. To avoid artifacts in the filtering phase, ES artifacts with a mean duration of 24.96 ± 1.37 ms were manually segmented from the raw data before applying the band-pass filter and running the spike detection algorithm. Moreover, an ES trial was determined as a significant stimulation, when the firing rate before and after the stimulation along the six stimulation pulses were statistically different. To this effect, data normality was checked with the Kolmogorov–Smirnov test and statistical differences were established applying paired sample *t*-tests with a significance level of 5% using Origin (Microcal Software, United States).

Additionally, the current measurement circuit inside the headstage allowed the measurement of the delivered current during ES (I_del_), which enabled also the calculation of the injected charge (Q_inj_) after the time integration of I_del_, and the calculation of the charge density (Q_d_) considering the ESA of the stimulating electrode, which was 576 μm^2^, as only 16-BiMEA probes were used during ES experiments. I_del_, Q_inj_, and Q_d_ were then calculated for both cathodic and anodic phases.

### Signal Analysis

Raw data were subjected to offline post-processing methods using self-written MATLAB (Mathworks Inc., United States) programs.

#### Filtering

As suggested by Quian Quiroga (2009), zero-phase filtering stages were used to obtain high and low frequency content. Raw data were filtered using 6th order Butterworth band-pass (high and low pass cutting frequencies of 100 Hz and 3 kHz) and a low-pass (cutting frequency of 100 Hz) filters to extract action potentials and LFP, respectively. Additionally, a Notch filter with a cutting frequency of 50 Hz was applied to the low-pass filtered signals, in order to eliminate the power line noise. Moreover, Fourier analysis was carried out to extract the frequency components of LFPs.

#### Spikes Analysis

To determine whether or not a peak was an action potential (spike), an algorithm based on the search of spikes whose inter-spike intervals were equal or greater than 3 ms (Rey et al., 2015), whose amplitude would surpass a threshold based on the absolute median deviation of the band-pass filtered signal (Quiroga, 2004), and whose prominence (MATLAB, Mathworks Inc., United States) was at least six times the absolute median deviation was implemented. The firing rate of the detected spikes was computed using histogram bins every 1 s, 500 ms, or 100 ms along the desired recording, thereby obtaining the count of spikes per bin depending on the spike count resolution needed. Such count was then normalized to obtain a firing rate in spikes per seconds, meaning Hz.

## Results

### Recording With the BiMEA Probes

The feasibility of using the penetrating BiMEAs for recording retinal activity is shown for wildtype and degenerated *rd10* retina.

#### Recording at Different Depths Inside Wildtype Retina

**FIGURE 4:**
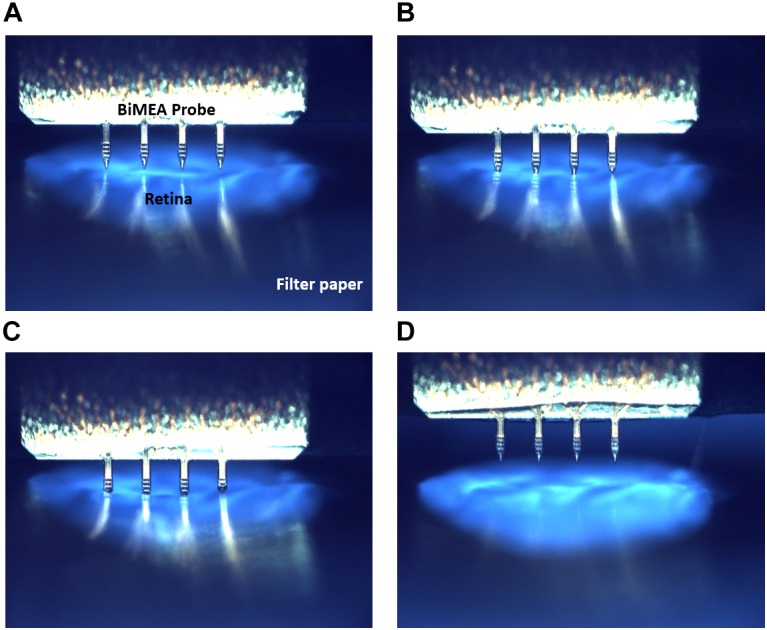
BiMEA insertion into the retina. Optical images showing four different insertion steps. First the BiMEA shanks are at the surface of the tissue before insertion **(A)**, then the tips of the shanks are driven into the tissue **(B)**, followed by the step-wise insertion of the shanks in **C**, and the final retraction of the probes at the end of an experiment in **D**. The tissue was illuminated from beneath, so that a contrast was generated between the filter paper carrying the tissue (dark blue), the retina (light blue), and the BiMEA shanks. A wildtype retina was used during this experiment.

As a first step in every experiment, the BiMEA electrodes were positioned at different locations inside the tissue, enabling the recording of electrical activity at different depths (Z_x_) of the retina. The insertion of the shanks was carried out stepwise: first the shanks were moved close to a position nearby the surface of the retina, then the insertion was continued until the top electrodes of the recording shank had captured spikes. The penetration was performed without the assistance of an optical system, but was assessed by observing the tip of the shanks through the glass ring with naked eyes and the electrical activity recorded by the electrodes. The insertion of the shanks was further confirmed in a dummy experiment (Figure 4). Here, the medium was extracted from the perfusion chamber to avoid the refraction of light due to the watery medium, leaving a semi-hydrated retina that was illuminated from beneath, so that a strong contrast between the carrier paper holding the tissue and the shanks was established for imaging.

**FIGURE 5:**
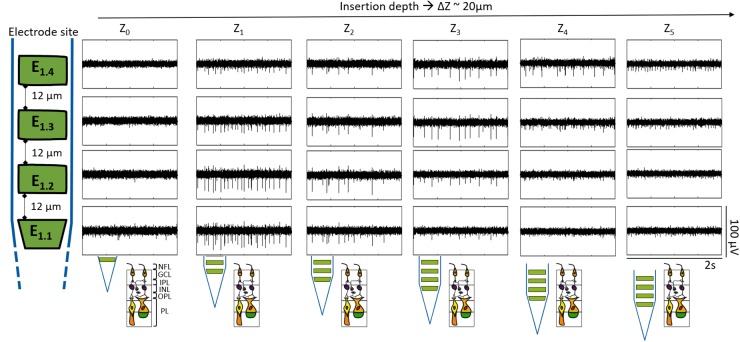
Recording at different depths inside wildtype retina. The boxes display segments of 2 s of the band pass filtered recordings for one shank. Each row corresponds to each one of the four electrodes of the same shank as indicated by the sketch at the left, and each column represents a different insertion depth (Z_0-5_) inside the retina, corresponding Z_1_ to 21.6 μm, Z_2_ to 39.7 μm, Z_3_ to 61 μm, Z_4_ to 80.8 μm, and Z_5_ to 100.7 μm in reference to Z_0_. The depth difference among each Z (ΔZ) was approximately 20 μm. Likewise, the expected shank position along the different depths within the retina is portrayed at the bottom.

In all experiments, the retina was penetrated from the nerve fiber layer/ganglion cell layer (NFL/GCL). In this part of the retina, action potentials are generated in somas and axons of RGC, while neurons located in deeper retinal layers do not fire action potentials. While penetrating the tissue, fast voltage deflections in the form of spikes were first recorded at the lowest of the electrodes. Spikes were observed at progressively higher electrodes when shanks were moved deeper into the retina (Figure 5). In this manner, the spontaneous activity (SA) of a wildtype retina was followed along a 100 μm trajectory inside the tissue. The electrical activity was first noticed by E_1.1_, which captured low amplitude peaks (≤18 μV), indicating that the bottom electrode of the shank was nearby the retinal surface. In this way, further depths at Z_1_ (21.6 μm), Z_2_ (39.7 μm), Z_3_ (61 μm), Z_4_ (80.8 μm), and Z_5_ (100.7 μm) corresponded to the position of the bottom electrode (E_1.1_) inside the retina with respect to Z_0_ (0 μm).

At Z_1_ spikes were detected in all four electrodes, however, the spike amplitude was higher at the bottom electrodes E_1.1_ and E_1.2_, with peak heights of 28.52 ± 5.08 μV and 31.83 ± 3.19 μV accordingly (see Figure 5 and Supplementary Table 1), thereby indicating that they were within the NFL and GCL. In such a way, the spike amplitude of the peaks detected by the electrodes increased while they entered the superficial layers of the tissue (NFL and GCL) and decreased as they penetrated deeper inside the retina. In this way, knowing that the distance from the top to the bottom electrode was 100 μm, considering that at Z_3_ E_1.3_ and E_1.4_ recorded the spikes with the highest amplitudes (see Supplementary Table 1), and taking into account that the summed thickness of the NFL, the GCL, and the inner plexiform layer (IPL) is around 70 μm (Dysli et al., 2015), it was then expected that the two top electrodes (E_1.3_ and E_1.4_) were in between the GCL and the NFL, that E_1.2_ was reaching the IPL, and that the bottom electrode (E_1.1_) was at the end of the IPL and reaching the inner nuclear layer (INL) of the retina (see bottom sketch in Figure 5). Consequently, no action potentials were captured with E_1.1_ and E_1.2_ at Z_4_, which were expected to be between the outer plexiform layer (OPL) and the INL. In contrast, the upper electrodes E_1.3_ and E_1.4_, which had moved further into the IPL and GCL were still recording spikes. Finally, at Z_5_, only low amplitude peaks (≤16 μV) were detected by the upper electrodes, meaning they were already beyond the RGCs.

**FIGURE 6:**
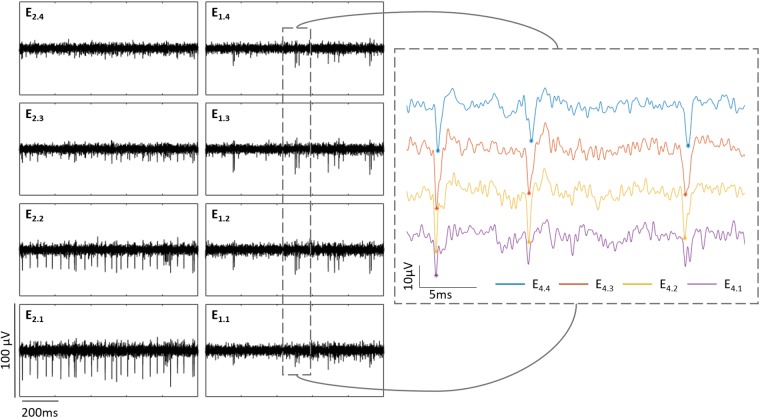
Shanks at different depths inside the retina. The boxes at the left show the electrical activity of a wildtype retina captured by two shanks at Z_2_ (42.8 μm) inside the retina. Each column represents each shank and each row an electrode along the shank. The graph at the right zooms the activity captured in shank one, depicting in purple E_1.1_, in yellow E_1.2_, in red E_1.3_, and in blue E_1.4_. The peaks detected as spikes in this extract are marked with an asterisk of the corresponding color.

It is important to notice that in cases when the explanted tissue was not completely flat on the PDMS pillow, the shanks did not contact the tissue at the same depth. The latter can be seen in Figure 4 and is also observed during the electrical recordings. For example in Figure 6, the electrical activity captured by two shanks at Z_2_ (42.8 μm) is shown. While the spiking activity of the vertical column in shank one is barely captured by the bottom electrode (E_1.1_) but detected by the upper electrodes (E_1.2_, E_1.3_, and E_1.4_), the bottom electrodes in shank two (E_2.1_ and E_2.2_) are the ones recording the action potentials of RGCs. The latter reveals that even though both shanks were inside the tissue, they were actually at different depths. Thus, when the Z positions were set, these corresponded to the shank whose electrodes captured the first spikes of the recording. Hence, the Z positions for the recordings displayed in Figure 6, were taken according to the first shank.

Furthermore, having shanks with multiple recording sites allowed us to follow the activity of a group of cells within a same vertical column inside the tissue. As exhibited in Figure 6, the same spikes with different amplitudes were captured by the electrodes of a same shank (depending on how deep inside the tissue they were), what indicates that whenever the bottom electrodes were not detecting any more spikes, it was because they had already passed the GCL, however, the activity of the cells present in that vertical column where the shank was located, was still recorded by the upper electrodes.

#### Recording Responses to Treatment With High Extracellular Potassium Concentration

**FIGURE 7:**
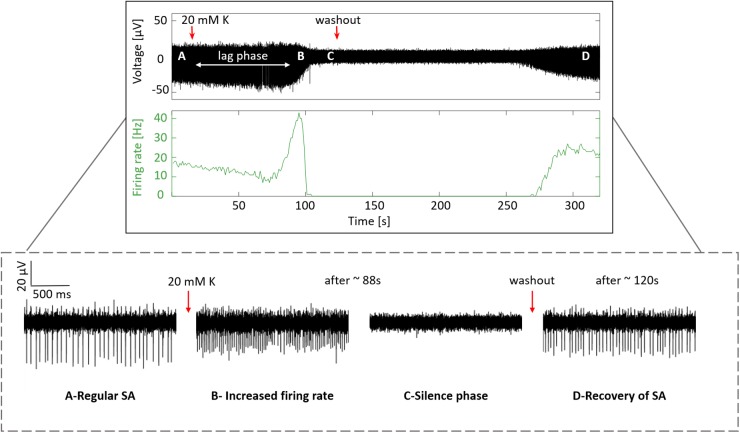
Response to treatment with high potassium. The top box displays the recording of the complete experiment, indicating with red arrows the application time of 20 mM K^+^ and the time when the washout with regular medium was started. The second top box shows the firing rate along the experiment with bin counts every second. The four plots inside the dashed box at the bottom show a zoom of the four phases distinguished during the experiment. **(A)** Regular SA captured at the beginning, **(B)** increased firing rate after the application of 20 mM K^+^, **(C)** silence phase, and **(D)** recovery of SA upon washout.

In order to confirm that the signals recorded previously indeed reflect physiological activity of RGCs in the form of action potentials, a wildtype retina was subjected to an increased extracellular K^+^ concentration, allowing us to observe a physiological retinal response to changes in the extracellular ionic concentration. A recording of one electrode site displaying the spiking activity and the firing rate along a complete experiment is shown in Figure 7, where four phases exhibiting the response to the treatment with high K^+^ were distinguished. First, regular SA with a firing rate of ∼17 Hz was detected (Figure 7A), then after the application of 20 mM K^+^ a lag phase was observed, followed by a transient increase in the firing rate with a spike count up to ∼40 Hz (Figure 7B) and a spikeless silent phase (Figure 7C), which was ended upon washout, thereby permitting the recovery of the spiking activity with a firing rate of ∼23 Hz (Figure 7D).

This behavior is in full agreement with our current understanding of how action potentials are generated. The increase in the external K^+^ concentration shifted the Nernst potential for K^+^ and, therefore, the membrane potential of RGCs to more positive values. This depolarization increased the firing rate of the recorded cells (Figure 7B). Continuous depolarization of the cells finally induced a depolarization blockade concomitant with a silence phase (Figure 7C), during which no action potentials could be fired because voltage-activated Na^+^ channels did not recover from inactivation. Afterward, when the extracellular K^+^ concentration was reduced with the perfusion of regular medium, the SA recovered.

#### Recording Responses to Light Stimulation

**FIGURE 8:**
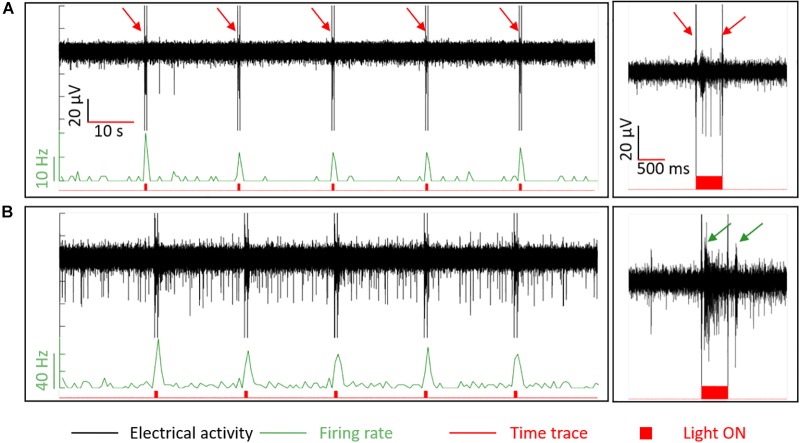
Responses to optical stimulation in wildtype retina. Light stimuli with an ON period of 500 ms every 15 s were used to stimulate the retina optically. In the first column, recordings of ON and OFF responses are shown. Plots at the second column correspond to a 3 s extract of the complete recording shown at the left. In **(A)**, the response of a transient and a sustained ON cell. In **(B)**, the bursting activity of two different cells (ON and OFF cells) are pointed out by the dark green arrows. Traces in black represent the electrical recordings (μV), in light green the firing rate with normalized bin counts every 500 ms [spikes/second (Hz)], and in red the corresponding time trace. The red filled-bumps match the time when the light stimuli were ON. The red arrows in **(A)** point out high amplitude peaks at the onset and offset of the light pulses, which are electrical artifacts induced by light seen in all the recordings when using light.

In order to confirm that the retinal integrity was preserved upon penetration by BiMEA electrodes, responses of a wildtype retina to optical stimulation were recorded with the penetrating BiMEAs. Light stimuli were 500 ms long and were repeated every 15 s (see Figure 8). Light-induced artifacts were observed in the recordings phase-locked with the ON-OFF switching of the light stimuli (pointed with red arrows in Figure 8A, but present in all shown examples). In principle, responses to light steps can be ON (increased spike frequency at light onset), OFF (ditto at light offset) or ON-OFF and can be transient or sustained. As retinas were prepared under ambient light and therefore not optimized for recording light responses, we did not attempt to document all response types, but rather show in a few examples that light responses could be recorded with the type of electrodes used here.

The recording in Figure 8A shows a low SA (<1 Hz), which was increased up to 20 Hz by a short burst of low amplitude peaks (∼15 μV) and a sequence of higher amplitude spikes (∼38 μV) that lasted until the end of the light stimulus, indicating the presence of a transient and a sustained ON cell recorded by this electrode. In the case of Figure 8B, a burst of spikes during the light stimulus (ON cell, first arrow) and a short burst after the offset (OFF cell, second arrow) could be observed. These recordings demonstrate that the retina could still respond to light after the penetration, indicating that tissue damage was minimal.

**FIGURE 9:**
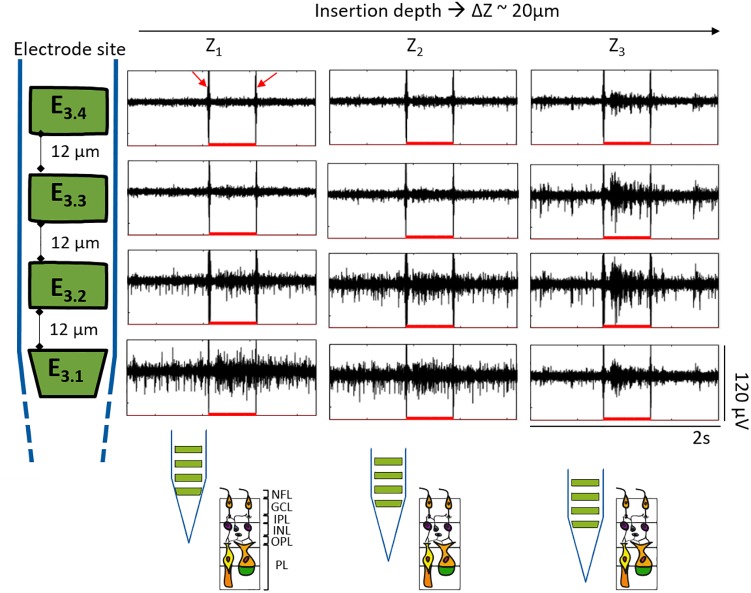
Responses to optical stimulation at different retinal depths. Segments of 2 s showing the electrical recordings during light stimulation. Each row corresponds to each one of the four different electrodes of the same shank as indicated by the shank sketch at the left. Insertion depths from Z_1_ to Z_3_ correspond to each column. The depth difference among each Z (ΔZ) was approximately 20 μm. The red filled-bumps depict the time where the light stimuli were ON (500 ms). Likewise, the expected shank position along the different depths within the retina is portrayed at the bottom.

Responses to light were also used in the experiments to assess the vitality of wildtype retinas while penetrating the tissue, ratifying that the same group of cells at different retinal depths were being recorded. Figure 9 exhibits an example of the follow-up performed to the ON cells captured in Figure 8B, showing the same optical response for Z_1_ (20.9 μm), Z_2_ (42.5 μm), and Z_3_ (63 μm). At Z_1_, the reaction to light was evident at the two bottom electrodes, indicating their proximity to the NFL and GCL. At Z_1_, the upper recording sites did not capture any action potential but displayed the electrical artifact induced by the stimuli (see red arrows). At Z_2_ the spiking activity became more visible for E_3.2_. Finally, at Z_3_ the action potentials were diminished in amplitude at E_3.1_, became notorious with higher amplitude peaks at E_3.2_ and E_3.3,_ and started to be captured by E_3.4_. Considering the responses to light captured at the upper electrodes, the reduced spikes captured by E_3.1_, and that the latter was at a depth of ∼63 μm inside the retinal tissue, it was an indication that the bottom electrode was entering the next retinal layer, the IPL, and that the retina was still vital. In this way, the BiMEA probes were placed in such a way that the bottom electrode would be located deep inside the retina, where spikes were barely captured or not captured at all, while the upper electrodes were still capable of recording the ongoing activity, so that further experiments, such as ES inside the retina, could be performed.

#### Recording From Degenerated Retina

**FIGURE 10:**
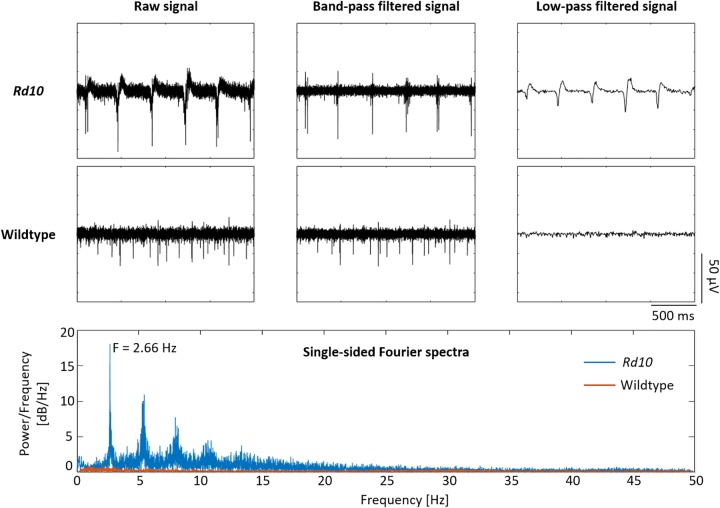
Comparison of wildtype and *rd10* recordings. *Rd10* and wildtype recordings are shown in the first two rows. The raw signal of *rd10* exhibits bursting activity that coincides with the presence of low frequency oscillations, which can be seen in the raw and low-pass filtered signals. Wildtype activity shows only the presence of stochastic spikes. At the bottom box, the single-sided Fourier spectra expose a main frequency component of ∼2.66 Hz for *rd10* LFPs, while none were encountered for the wildtype.

Given that it was possible to record the electrical activity of a vital wildtype retina with the penetrating BiMEAs, these were also used to capture the activity of retinas with photoreceptor degeneration. To this end we employed retinas of *rd10* mice, a mouse line that is considered a suitable model for the human disease RP. When positioning the shanks inside the degenerated tissue, a rhythmic activity was recorded (Figure 10). This pathological activity is not observed in wildtype retinas. In *rd10* mouse retina, the oscillations are commonly observed (Goo et al., 2011a; Stasheff et al., 2011; Jae et al., 2013; Biswas et al., 2014; Haselier et al., 2017) but may come and go throughout the recording (Biswas et al., 2014). Oscillations can be observed in the raw recording as well as in the low-pass filtered signal, the LFP. Fourier analysis showed a main oscillating frequency of the LFPs around 2.6 Hz, while none were present in the wildtype. Moreover, an inherent bursting activity often phase-locked to the negative deflection of the LFP was observed in comparison to the stochastic spiking activity of a healthy retina (Figure 10).

**FIGURE 11:**
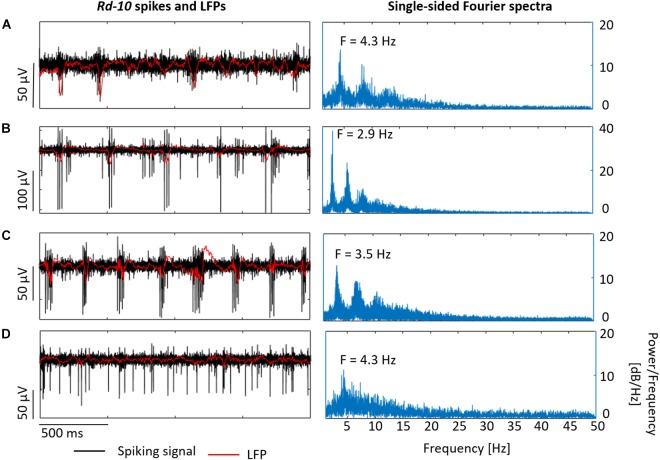
Recording different LFPs in *rd10* retinas. *Rd10* spikes and LFPs in **(A–C)** exhibit the electrical activity of the same retina at different x-y locations, displaying in black the band-pass filtered signal (spiking signal) and in red the low-pass filtered signal (LFP). At the right column, single-sided Fourier spectrum is shown for each case. In the case of **(D)**, which shows the activity of a different sample, the typical *rd10* electrical behavior was not evident, but oscillatory components in the LFPs were exposed by the corresponding Fourier spectrum.

Spike bursts and LFPs with frequencies ranging from 2.6 to 4.3 Hz were also observed at different x-y locations within the same retina (Figure 11A–C). These oscillatory frequencies agree well with the typical range of 3–6 Hz reported by Biswas et al. (2014). Moreover, in some *rd10* samples (Figure 11D), spike bursts and oscillations were not evident in the LFP, however, the Fourier spectrum revealed an increased power for frequencies ranging between 2.5 and 7 Hz and peak frequencies around 4.3 Hz, showing that an oscillatory component was present in the recorded activity even when not obvious during the live recordings.

**FIGURE 12:**
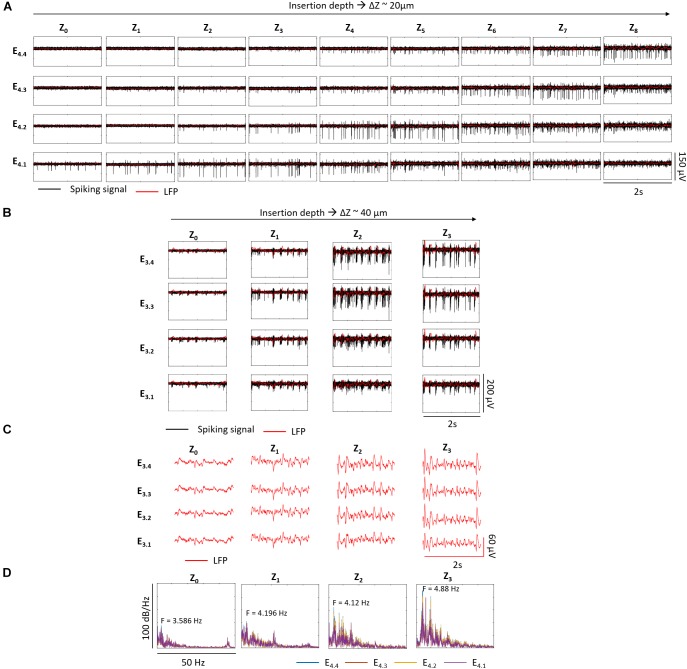
Recording at different depths inside *rd10* retina. In A, the electrical activity of one shank along Z_0_–Z_8_ with Z steps of ∼20 μm, corresponding Z_1_ to 21 μm, Z_2_ to 42.6 μm, Z_3_ to 63.8 μm, Z_4_ to 82.3 μm, Z_5_ to 101.7 μm, Z_6_ to 121.7 μm, Z_7_ to 141 μm, and Z_8_ to 162.1 μm with respect to Z_0_ is shown. Each row corresponds to each one of the four electrodes of the same shank as indicated and each column represents a different insertion depth (Z_x_) indicated. B shows the electrical activity of a second *rd10* retina with Z steps of ∼40 μm. Z_1_ corresponds to 41.5 μm, Z_2_ to 80 μm, and Z_3_ to 122.9 μm with respect to Z_0_. The black traces show the spiking signal and in red the LFPs. In C the LFPs, and in D the corresponding single-sided Fourier Spectra at each depth for all the electrodes of the shank displayed in **B,C**. The peak frequency of the low frequency oscillations range between 3.586 and 4.88 Hz along Z_0-3_.

We observed some differences between recordings from wildtype and *rd10* retinas. During penetration of the wildtype retina with the shanks, the recording with the highest spike amplitude moved along the shank from the bottom to the top electrode (see e.g., Figure 5). A similar effect was observed in *rd10* retina, however, the effect was less pronounced and decent spike recordings could be observed over more penetration steps than in wildtype retina (see Figure 12A,B and Supplementary Tables 2, 3). Assuming that the highest spike amplitude was recorded by the electrode which was closest to the RGCs, the recordings at Z_8_ and Z_3_ indicated that the top electrodes E_4.4_ and E_3.4_ were in between the GCL (see Figure 12A,B accordingly). At this last depth, the top electrodes captured higher amplitude spikes of 54 ± 11.14 μV and 69.07 ± 23.44 μV, while the bottom electrodes E_4.1_ and E_3.1_ had gone through deeper layers. Considering that the thickness of the retina in adult *rd10* mice is ∼100 μm (Pennesi et al., 2012) and that each bottom electrode had gone through ∼100 μm (E_4.1_ from Z_3_) and 81.4 μm (E_3.1_ from Z_2_) inside the retina, it was expected that E_4.1_ was reaching the end of the tissue at the outer margin of the INL. While no spikes are generated at this depth, the bottom electrodes captured the same spikes as the upper electrodes, albeit at lower amplitudes of 30 ± 6.81 μV (E_4.1_) and 44.43 ± 9.18 μV (E_3.1_). Finally, the average maximum spike amplitude in wildtype retina was ∼39 μV, while in *rd10* it was ∼100 μV (see Supplementary Table 4).

It was possible to do a follow up of the pathologic rhythmic activity of the degenerated *rd10* tissue along different depths. While the typical spike bursts and oscillations were not obvious in the recordings exhibited in Figure 12A, Fourier analysis showed an increasing power of oscillatory components ranging between 2.5 and 7 Hz as deeper distances within the retina were achieved, with a peak frequency of 4.3 Hz at Z_8_ (see Supplementary Figures 2A,B). A clearer behavior was captured when Z steps of ∼40 μm were performed (see Figure 12B–D). Here, spike bursts coupled with low frequency waves were noticed from Z_0_ until Z_3_, with an oscillatory component that increased in power and whose peak frequency was shifted from 3.586 to 4.88 Hz as the electrodes advanced deeper inside the retina (see Figure 12D).

### Electrical Stimulation and Recording

After validating the feasibility of using the BiMEA probes for recording retinal activity and accessing the different retinal layers for both wildtype and *rd10* retinas, we set out to electrically stimulate neurons of the inner retina with the lowermost electrode while at the same time record activity of RGCs with the upper electrodes. Following the insertion methodology, the shanks of the BiMEA probes were first placed inside the tissue until the recordings of at least one shank would indicate adequate penetration with higher amplitude spikes at the top electrodes and lower amplitude spikes or no spikes at the bottom electrode in deeper layers. Likewise, the vitality of the tissue was assessed by the recording of SA and, in the case of wildtype retinas, responses to light stimulation. Afterward, ES in a voltage-controlled mode was carried out, using the bottom electrode (E_X.1_) of the selected shank as the stimulating electrode, and the rest as recording electrodes. In this way, the shank carrying the stimulating electrode would be referred as the stimulated shank, and the others as non-stimulated shanks. Different sets of stimulation parameters termed ES-1 to ES-6 were chosen and the stimulation efficiency ESE was determined.

**FIGURE 13:**
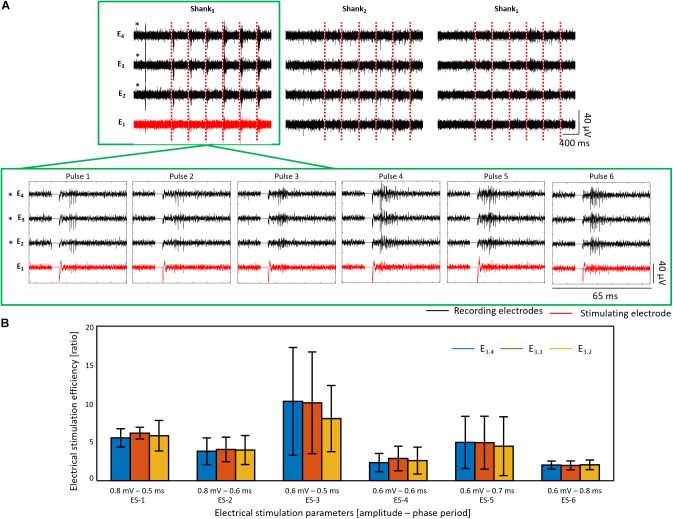
Electrical stimulation and recording in wildtype retina. The electrical activity after six consecutive biphasic pulses (first cathodic) with 0.6 mV in amplitude and a phase period of 0.5 ms (ES-3) every 20 s at E_1_ of shank 3 is shown for a wildtype retina in **(A)**. Recording extracts of the first second before electrical stimulation (ES) followed by the electrical activity of the first 400 ms after each electrical stimulus are displayed for each shank (column) and each electrode (row). The ES pulses are represented by red dashed lines, and the activity captured by the recording and stimulating electrodes is displayed in black and red, respectively. Additionally, a zoom of the electrical activity captured 15 ms before and 25 ms after each ES pulse (stimulation artifact shown as a line of 0 V) is shown for the shank of interest, pointed out with a green frame. Electrodes with a significant stimulation (*p* < 0.05) are denoted with an asterisk (^∗^). In **(B)**, the electrical stimulation efficiency (ESE) for six different ES parameters are shown for the same retina used in **(A)**. For this experiment, shank 4 is not shown due to non-working electrodes.

ES was first tested in wildtype retinas, which exhibited a burst of spikes when a reaction to an electrical stimulus was present. Figure 13A shows an example of a wildtype retina (for vitality of sample and positioning of electrodes see Supplementary Figure 3A) stimulated with six ES pulses using ES-3 parameters (0.6 mV – 0.5 ms) every 20 s. The bursting reaction was evoked pulse by pulse, showing a successful stimulation with an activation effect on RGCs, as significant firing rate differences (*p* < 0.05) with ESEs higher than one were revealed for the three recording electrodes (E_3.4_, E_3.3_, and E_3.2_) of the stimulated shank (see recordings inside the green frames in Figure 13A,B). Successful stimulation was observed for six different stimulation parameters with ES-3 yielding the highest ESE between 8.04 ± 4.29 and 10.27 ± 6.97 (see Figure 13B).

Likewise, mean ESEs between 1.26 ± 1.40 and 2.5 ± 2.35 were detected in shank 2, however, the electrical responses captured by the electrodes of this non-stimulated shank were not constant along the six stimuli. In this way, a non-significant stimulation was produced for cells recorded at shank 2, while responses to the stimuli were barely captured from cells recorded by shank 1. Moreover, a significant reduction of the firing rate with ESEs lower than one was also observed during ES-1 (in E_2.4_) and ES-4 (in E_2.4_ and E_2.3_) in recording electrodes of non-stimulated shanks, exposing therewith an inhibition effect after ES (see Supplementary Table 5).

**FIGURE 14:**
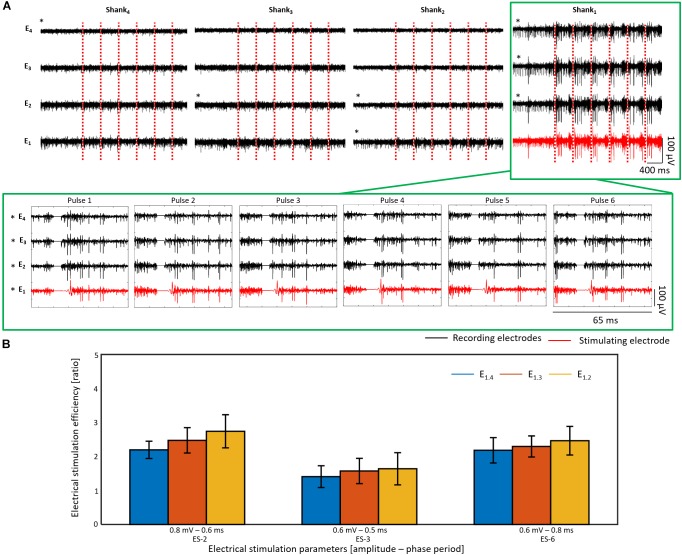
Electrical stimulation and recording in *rd10* retina. In **(A)** the electrical activity after six consecutive biphasic pulses (first cathodic) with 0.8 mV in amplitude and a phase period of 0.6 ms (ES-2) every 20 s is shown for an *rd10* retina. Recording extracts of the first second before electrical stimulation (ES) followed by the electrical activity of the first 400 ms after each electrical stimulus are displayed for each shank (column) and each electrode (row). The ES pulses are represented by red dashed lines, and the activity captured by the recording and stimulating electrodes is displayed in black and red, respectively. Additionally, a zoom of the electrical activity captured 15 ms before and 25 ms after each ES pulse (stimulation artifact shown as a line of 0 V) is shown for the shank of interest, pointed out with a green frame. Electrodes with a significant stimulation (*p* < 0.05) are denoted with an asterisk (^∗^). In **(B)**, the electrical stimulation efficiency (ESE) for three different ES parameters is shown for the same retina used in **A**.

Figure 14A exhibits the electrical responses of an *rd10* retina (see SA of sample before ES and positioning of electrodes in Supplementary Figure 3B) stimulated with six consecutive pulses every 20 s using ES-2 (0.8 mV and 0.6 ms). Here, the presence of at least two different cells in the recordings of shank 1 was noted, as two different spike amplitudes stood out, exposing thereby responses that comprised a mixture of discontinuous spikes with an increased firing rate and bursts of action potentials. Similarly to wildtype retinas, successful stimulation was observed at the three upper electrodes of the stimulating shank (*p* < 0.05), however, the mean ESEs of the electrodes was lower than in wildtype, between 2.2 and 2.7. Significant stimulations were also obtained using ES-3 and ES-6 on this retinal sample, yet the ESEs were lower than for ES-2, ranging between 1.4 and 2.2 (see Figure 14B and Supplementary Table 6). Differences in the ESE between wildtype and *rd10* retinas were further confirmed when comparing the average ESE of the stimulated shanks during successful stimulations. In this way, ES-1, ES-2, and ES-3 proved to evoke significantly higher ESEs in wildtype than in *rd10* samples (see Supplementary Figure 4A).

Unlike to the electrical responses evoked in wildtype retinas, successful stimulations eliciting the activation of RGCs in the recording electrodes of non-stimulated shanks were detected in *rd10* retinas, as revealed by electrodes E_4.4_, E_3.2_, E_2.2_, and E_2.1_ (see Figure 14A and Supplementary Table 6). While the recordings before ES suggested that the bottom electrodes of the non-stimulated shanks had reached the GCL (E_4.1_) and the NFL (E_3.1_ and E_2.1_) in the *rd10* sample (see Supplementary Figure 3B), electrical responses were also captured in the upper electrodes with low amplitude spikes. The fact that E_4.4_, E_3.2_, and E_2.2_ presented significant firing rate differences can be attributed to the sensitivity of the ESE when a low SA (higher than 0 and lower than 1 Hz) is being captured, since the ratio would calculate an ESE higher than 1 even when one spike is detected after ES, and would rise extremely if the activity before ES is slightly higher than 0 Hz. Nevertheless, consistent and significant electrical responses generating firing rate increases were observed in shank 2 during the three different ES parameters tested on the sample (see Supplementary Table 6). Moreover, considering that the inter-shank distance was 190 μm, the stimulation of distant cells with respect to the stimulating electrode was unveiled for *rd10* retinas.

Furthermore, measurements of the delivered current during ES exposed minimum cathodic currents of -4.62 ± 2.92 μA and maximum anodic currents of 6.43 ± 4.04 μA for the generation of successful stimulations on both wildtype and *rd10* samples. Depending on current amplitude and stimulus length, cathodic and anodic charge densities between -686.10 ± 304.16 μC/cm^2^ and 555.69 ± 308.41 μC/cm^2^, considering an ESA of 576 μm^2^ (see Supplementary Table 7 and Supplementary Figures 4B,C) triggered stimulation of cells. Hence, our stimulation parameters lie in the range also employed by other researchers (Stett et al., 2000; Suzuki et al., 2004; Jalligampala et al., 2017; Corna et al., 2018). In addition, despite the higher cathodic but lower anodic charge densities encountered in *rd10* retinas during ES-3 and ES-6, no proportional relationship between higher cathodic currents and higher stimulations efficiencies were found, what can be explained by the high variability of the delivered currents during ES (see Supplementary Figures 4B,C).

Finally, reproducibility of the evoked responses was confirmed after testing six different stimulation parameters in three different wildtype retinas, obtaining significant stimulations in two out of three retinas after applying ES-1, ES-2, ES-4, ES-5, and ES-6, and in one retina using ES-3. In contrast, *rd10* retinas showed successful stimulations after ES-1, ES-3, and ES-6 in one out of two retinas, and after ES-2 in two out of two retinas.

## Discussion

### Multi-Site Penetrating MEAs for Retinal Applications

With the aim to achieve a closer proximity to target neurons and reduce charge densities during ES for retinal implants, penetrating electrodes have been investigated by several research groups in the form of pillars or protuberant 3D electrodes (Yanovitz et al., 2014; Bendali et al., 2015; González Losada et al., 2017; Flores et al., 2018). In order to amplify and complement such efforts into a bidirectional communication between a prosthetic device and retinal cells, multi-shank and multi-site penetrating MEAs (Michigan-like probes), which have been used mostly as intracortical neural interfaces (Weltman et al., 2016), were tested in this work to prove the feasibility of simultaneous intraretinal electrical recording and stimulation. Penetrating pillars or protuberant electrodes are typically used for unidirectional communication, i.e., ES, while to our knowledge, the application of multi-site penetrating electrodes for a bidirectional communication with simultaneous ES and recording of the retina has not been published before. Even healthy retina is only 200 μm thick, much thinner than the neocortex. Upon photoreceptor degeneration, the remaining retina is only 100 μm in thickness. Hence, in the design of our device we carefully optimized size and distance of the electrodes to match these restrictions.

The use of penetrating shanks with multiple electrode sites made it possible to place the bottom electrodes of the shanks (later on used as stimulating electrodes) in deeper retinal layers, while at least one of the upper electrodes came in close proximity to the GCL, to continuously record the spiking activity of the retina. Recordings of spontaneous electrical activity, as well as meaningful physiological responses to optical stimulation and changes in the extracellular ionic concentrations proved that the recorded spikes originated from RGCs, showing in turn the vitality of the tissue during the intraretinal recordings. Moreover, the penetrating MEAs were also capable of crossing different retinal depths in degenerated *rd10* retinas while recording the typical pathologic rhythmic activity present in *rd10* mice (Goo et al., 2011a; Stasheff et al., 2011; Jae et al., 2013; Biswas et al., 2014; Haselier et al., 2017). After proving the capabilities of the penetrating BiMEAs to access different retinal layers while recording the retinal activity, ES of neurons of the inner retina was carried out using only the bottom electrodes as stimulating electrodes. The upper electrodes of the stimulated shank, located close to the GCL, were then used together with the electrodes of non-stimulated shanks as recording electrodes. In this way, successful electrical responses in both wildtype and *rd10* retinas were captured during simultaneous intraretinal recordings. Bursting reactions to different electrical stimuli were exposed for wildtype retinas, while bursting activity as well as discontinuous spikes were observed for *rd10* retinas. Additionally, lower ESEs were revealed in *rd10* in comparison with wildtype retinas, agreeing with the ES behaviors reported by Haselier et al. (2017) using planar MEAs. While electrical recording and stimulation of retinal neurons can be also achieved with planar MEAs, multi-site penetrating probes allow the possibility to record from the same neuronal column being stimulated. In this way, it was noted that in wildtype retinas ES evoked excitatory responses confined to the neurons within the neuronal column along the stimulated shank. A device that can control the stimulating current and simultaneously record the success of ES could enable a bidirectional communication that provides feedback about the success of ES, capture the presence of abnormal retinal activity, and in principle perform an autonomous calibration of stimulating parameters.

In addition, current measurements during ES exposed injected currents and charge densities within the range of subretinal ES thresholds (100–900 μC/cm^2^) when using small electrode sizes (∼706 μm^2^) on *rd10* retinas, as recently reported by Corna et al. (2018). The charge densities revealed here surpass the thresholds reported by Yanovitz et al. (2014) and Bendali et al. (2015) when using penetrating pillar and protuberant electrodes inside the retina. However, optimization of stimulation modes, such as current- and charge-controlled stimulation as well as ES parameters for the BiMEA probes were beyond the scope of the present study and must be addressed in future studies.

We observed some differences between wildtype and *rd10* retina. In wildtype retina using 20 μm steps, we could clearly observe how the electrode that proved optimal for spike registration changed when the shank was inserted deeper into the retina. In *rd10* retina, this was less clear. Mechanical differences between wildtype and *rd10* retinas, such as an increased stiffness (Hamon et al., 2017), might have interfered with the insertion of the penetrating shanks into the *rd10* retina, suggesting that a higher insertion force might be needed when approaching the degenerated tissue. Likewise, in comparison with wildtype retinas, the decreased resistivity in *rd10* retina (Wang and Weiland, 2015) could explain higher spike amplitudes during *rd10* recordings and the presence of retinal spikes at the bottom electrodes when the top electrodes of a shank indicated to be nearby the GCL of the retina. The lower resistivity of the degenerated tissue could also explain the fact that for some stimulation parameters, higher charge densities were achieved in *rd10* retinas in comparison with wildtype, what could have elicited electrical responses in distant neurons. Hence, comparing the results between wildtype and *rd10* retina suggests that the successful stimulation of a narrow group of neurons is possible, however, the stimulation parameters should be tuned for each type of retina.

### Design Considerations and Future Penetrating BiMEAs

When compared to planar MEAs, the use of penetrating MEAs is certainly a more invasive method, and this is why efforts must be focused on the optimization of design and materials to minimize the potential damage of a penetrating intraretinal implant.

On one side, the design of such probes must consider the anatomy and microstructure of the retina. The first generation of BiMEA probes (12-BiMEAs) exhibited in this work, had a shank length of 1000 μm, which was reduced to 312 μm in a newer design (16-BiMEAs), coming closer to the total retinal thickness of approximately 200–220 μm in wildtype mice and 100–120 in *rd10* (Pennesi et al., 2012; Dysli et al., 2015; Li et al., 2018). Considering that region of interest for the penetrating MEAs inside the retina comprises from the NFL to the outer margin of the INL (∼100 μm), the shank length of future designs could be further reduced. Similarly, the selection of smaller electrodes (from 80 × 20 μm^2^ to 40 × 20 μm^2^) lead to the optimization of the shank width, which was reduced from 100 to 60 μm.

Additionally to the reduction of electrode dimension, a geometry modification of the stimulating BiMEA electrode (from rectangular to trapezoidal) helped to reduce the distance from the tip to the bottom electrode, thereby avoiding to pierce completely the retina during positioning of the electrodes. As smaller electrodes could reduce the dimensions of the penetrating shanks and increase spatial resolution, appropriate electrode materials that yield low impedances and high charge delivery capacities, such as IrO_x,_ PEDOT, or nanostructured Pt (Boehler et al., 2017), should be considered. In addition, different electrode configurations, like the use of a local return electrode, could be tested in order to narrow the scope of the ES (Weiland et al., 2016).

Furthermore, new materials that attenuate the mechanical and biological mismatch between the retinal tissue and the implant should be considered. Current planar retinal implants are based on flexible substrate materials such a polyimide, parylene-C, and silicone rubber (Weiland and Humayun, 2014), however, the growing generation of penetrating probes for retinal applications, including pillar electrodes and the one presented in this work, are mostly based on stiff materials like silicon (Yanovitz et al., 2014; Flores et al., 2018). Considering that the use of stiff materials can lead to glial responses and scar tissues hindering the long term functionality of neuronal implants (Weltman et al., 2016), in the future flexible and compliant penetrating retinal probes must be pursued. Therefore, in order to boost the potential use of bidirectional penetrating MEAs for retinal applications, further tests to investigate the mechanical properties and biological impact of such probes with respect to the retina are needed.

## Conclusion

This work unveiled the feasibility of using multi-shank and multi-site penetrating MEAs for retinal applications. In this way, different layers of the retina were accessed, offering at the same time the possibility to stimulate the inner retina and to follow-up the electrical activity along the same neuronal column by simultaneous recording of RGCs. Thus, the use of such systems could enable a bidirectional communication that provides feedback about the success of ES, captures the presence of abnormal retinal activity, and in principle could perform an autonomous calibration of stimulating parameters. While this proof of concept opens the door to potential intraretinal implants, it must be taken into account that it is an invasive technique and that the biological impact on the retina has not been established yet. Therefore, future bidirectional penetrating implants must focus on the assessment and reduction of potential damages to the retina, as well as on the development of flexible and complaint penetrating probes.

## Ethics Statement

All experiments were performed in accordance with the German Law for the Protection of Animals and after approval was obtained by the regulatory authorities, the Forschungszentrum Jülich and the Landesamt für Natur, Umwelt und Verbraucherschutz of the German federal state North-Rhine Westfalia.

## Author Contributions

WM, PW, FM, and AO conceived the idea and designed the experiments. SL fabricated the devices. VRM and JG performed electrical and biological experiments. VRM and SL performed the characterization of the electrodes. The manuscript was written through contributions of all authors. All authors have given approval to the final version of the manuscript.

## Conflict of Interest Statement

The authors declare that the research was conducted in the absence of any commercial or financial relationships that could be construed as a potential conflict of interest.
