# Supplementary material for: Toward a Bidirectional Communication Between Retinal Cells and a Prosthetic Device – A Proof of Concept
Source: Front Neurosci. 2019 Apr 30;13:367. doi: 10.3389/fnins.2019.00367 (PMC6502975; doi:10.3389/fnins.2019.00367)
Supplement: Supplementary file 1 [file Presentation_1.pdf]

Supplementary Material

1 Supplementary Figure 1

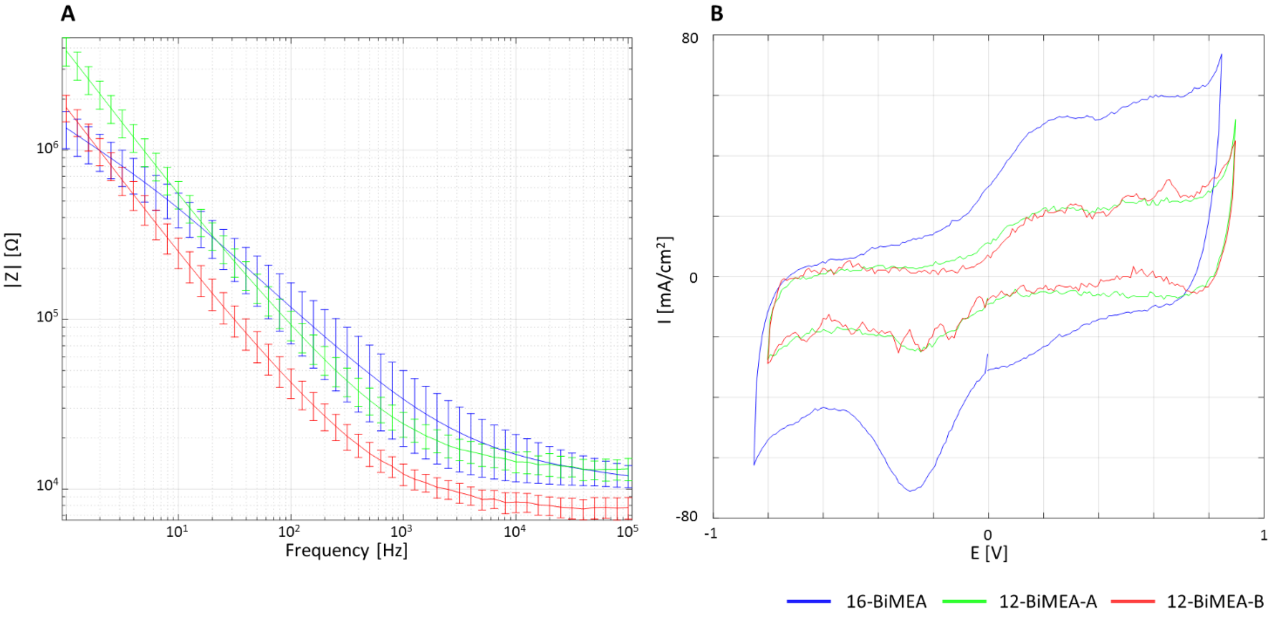

Supplementary Figure 1. Electrochemical properties of the BiMEA Probes. (A) Mean impedance spectra of the BiMEA probes showing the magnitude impedance (|Z|) along the frequency range of 1 Hz-100 kHz. (B) Average cyclic voltammetry (CV) curves, where I stands for current density and E for the electrode potential versus the Ag/AgCl electrode. In blue the 16-BiMEAs (N=4), in green the 12-BiMEA-As (N=3), and in red the 12-BIMEA-Bs (N=2).

2 Supplementary Table 1

Supplementary Table 1. Spike amplitude at different depths inside wildtype retina. Mean spike amplitude and standard deviation values of the detected spikes in the recordings shown in Figure 4 for each electrode in shank one along Z_{0-5} every ~20 µm inside the retina. The electrodes with higher amplitudes at a certain Z were marked in green, and a "-" was used to indicate that no spikes were detected.

Electrode	Spike amplitude at different insertion Depths [µV]					
	Z_0	Z_1	Z_2	Z_3	Z_4	Z_5
$E_{1.4}$	-	19.72 ± 1.02	21.47 ± 1.41	26.76 ± 3.61	26.43 ± 0.30	15.86 ± 0.54
$E_{1.3}$	-	23.05 ± 1.73	27.61 ± 2.49	31.91 ± 4.66	16.44 ± 0.22	15.79
$E_{1.2}$	-	31.83 ± 3.19	29.90 ± 8.21	21.22 ± 2.79	-	-
$E_{1.1}$	-	28.52 ± 5.08	24.87 ± 2.88	17.66 ± 0.93	-	-

3 Supplementary Table 2

Supplementary Table 2. Spike amplitude at different depths inside *rd10* retina. Mean spike amplitude and standard deviation values of the detected spikes in the recordings in Figure 12A for each electrode in shank four along Z_{0-8} every ~20 µm inside the *rd10* retina. The electrode with a higher amplitude at a certain Z was marked in green, and a “-” was used to indicate that no spikes were detected.

Electrode	Spike amplitude at different insertion Depths [µV]								
	Z_0	Z_1	Z_2	Z_3	Z_4	Z_5	Z_6	Z_7	Z_8
$E_{4.4}$	-	-	20.04 ± 1.78	19.96 ± 1.61	23 ± 2.60	24.70 ± 2.60	28.44 ± 3.61	37.57 ± 4.87	54.20 ± 11.14
$E_{4.3}$	18.11 ± 0.41	19.40 ± 1.40	22.12 ± 2.49	23.65 ± 2.82	24.23 ± 2,92	27.49 ± 3.56	43.68 ± 6.91	52.77 ± 12.49	33.50 ± 4.99
$E_{4.2}$	17.26 ± 1.95	16.64 ± 1.33	23.24 ± 2.76	37.68 ± 3.64	59.94 ± 15.74	50.16 ± 19.58	39.91 ± 9.60	33.67 ± 5.44	33.30 ± 5.23
$E_{4.1}$	32.54 ± 3.30	46.62 ± 9.26	52.30 ± 21.12	48.53 ± 21.40	39.75 ± 10.28	30.56 ± 5.50	30.26 ± 4.77	30.82 ± 4.67	30.48 ± 6.81

4 Supplementary Table 3

Supplementary Table 3. Spike amplitude at different depths inside *rd10* retina. Mean spike amplitude and standard deviation values of the detected spikes in the recordings in Figure 12B for each electrode in shank three along Z_{0-3} every ~40 µm inside the *rd10* retina. The electrodes with a higher amplitude at a certain Z were marked in green.

Electrode	Spike amplitude at different insertion Depths [µV]			
	Z_0	Z_1	Z_2	Z_3
$E_{3.4}$	16.1 ± 1.38	26.21 ± 5.70	53.06 ± 12.68	69.07 ± 23.44
$E_{3.3}$	17.74 ± 2.41	37.63 ± 10.95	59.65 ± 18.19	64.68 ± 21.86
$E_{3.2}$	18.27 ± 2.33	33.22 ± 7.21	41 ± 8.13	47.13 ± 12.03
$E_{3.1}$	22.68 ± 4.29	32.79 ± 6.94	38.55 ± 7.20	44.43 ± 9.18

5 Supplementary Figure 2

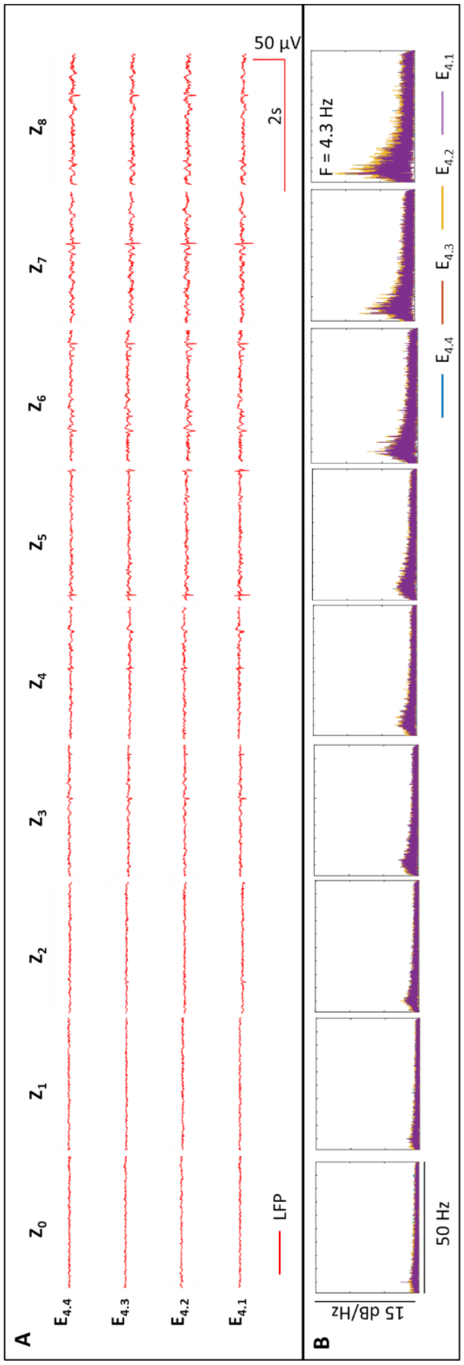

Supplementary Figure 2. Recording at different depths inside *rd10* retina. In (A) the local field potentials (LFPs) and in (B) the corresponding Fourier spectra at different depths from $Z_{0\text{-}8}$ with Z steps of ~ 20 µm, corresponding Z_1 to 21µm, Z_2 to 42.6 µm, Z_3 to 63.8 µm, Z_4 to 82.3 µm, Z_5 to 101.7 µm, Z_6 to 121.7 µm, Z_7 to 141 µm, and Z_8 to 162.1 µm with respect to Z_0. The data shown, is the counterpart of the recordings exhibited in Figure 12A.

6 Supplementary Table 4

Supplementary Table 4. Spike amplitude *rd10* versus wildtype retina. The maximum, minimum, mean, and standard deviation (SD) of the detected spike amplitude was calculated for electrodes positioned close to the ganglion cells, according to the depth and the spontaneous activity (SA) present. Experiments recording only the SA of RGCs without any stimuli and using only the 16-BiMEA probes with *rd10* and wildtype retina were selected.

Rd10 retina						
**Experiment**	**Depth**	**Electrode**	**Maximum amplitude [µV]**	**Minimum amplitude [µV]**	**Mean spike amplitude [µV]**	**SD spike amplitude [µV]**
Trial-1	Z_3 (122.9 µm)	$E_{3.4}$	142.78	28.82	69.07	23.44
Trial-2	Z_1 (84.3 µm)	$E_{2.2}$	48.63	17.73	33.15	8.45
Trial-3	Z_1 (187.1 µm)	$E_{1.2}$	86.92	23.25	30.91	8.39
Trial-4	Z_4 (82.3 µm)	$E_{4.2}$	85.27	19.10	59.94	15.74
**Average**			**90.90**	**22.22**	**48.27**	**14.00**
**Wildtype retina**						
**Experiment**	**Depth**	**Electrode**	**Maximum amplitude [µV]**	**Minimum amplitude [µV]**	**Mean spike amplitude [µV]**	**SD spike amplitude [µV]**
Trial-1	Z_4 (82.9 µm)	$E_{3.3}$	17.27	11.34	12.81	1.22
Trial-2	Z_4 (80.7 µm)	$E_{1.3}$	40.84	18.85	31.83	4.89
Trial-3	Z_3 (63 µm)	$E_{3.3}$	44.00	19.38	26.17	6.08
Trial-4	Z_1 (22.2 µm)	$E_{1.1}$	42.80	18.57	30.35	5.96
Trial-5	Z_1 (104.9 µm)	$E_{1.3}$	49.12	23.72	28.62	4.00
**Average**			**38.81**	**18.37**	**25.96**	**4.43**

7 Supplementary Figure 3

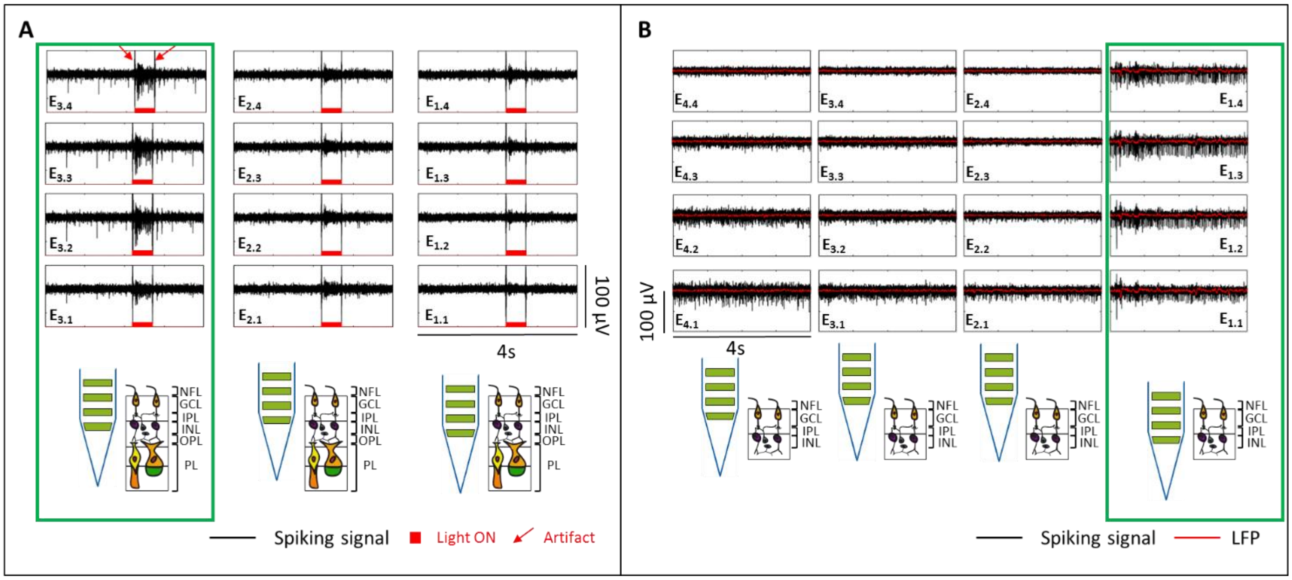

Supplementary Figure 3. Retinal vitality and positioning of electrodes before electrical stimulation experiments. Four seconds extracts showing the vitality of two different retinal tissues. Each column comprise the recordings of the four electrodes of each shank. In (A), the recordings of three shanks displaying the SA and the reaction to light stimulation of the wildtype retina shown in Figure 13A. The red filled-bumps depict the time where the light stimuli were ON (500 ms) and the red arrow show the light stimulation artifact. In (B), the SA (spiking signal in black) of the *rd10* retina shown in Figure 14A, and the low frequency signal (LFPs) in red. Likewise, the expected position of the

shanks in (A) and (B) is portrayed by the retina and shank schematics at the bottom. Additionally, the shank to be stimulated is pointed out with a green frame.

8 Supplementary Table 5

Supplementary Table 5. Significant stimulations in wildtype retina. The ESEs of each recording electrode along the six electrical pulses are displayed for the example shown in Figure 13. The average ESE and the corresponding standard deviation (SD) are also shown for the six ES parameters tested. Additionally, the electrodes that presented a significant stimulation (* = p<0.05) with a mean ESE >1 are marked in dark green, and those with a mean ESE <1 are marked with light green. “NA” stands for not applicable, indicating the cases where no activity was detected.

ES parameter	Electrical stimulation efficiency (ESE)											
ES-1 (0.8 mV - 0.5 ms)	Pulse	Shank3			Shank2				Shank1			
		3,4*	***3,3****	***3,2****	***2.4****	***2.3****	***2.2***	***2.1***	***1.4***	***1.3***	***1.2***	***1.1***
	p1	3.78	5.14	3.75	0.00	0.00	1.11	0.00	0.00	0.00	0.00	0.00
	p2	5.95	6.47	5.16	0.00	1.25	0.95	1.18	2.67	1.43	20.00	NA
	p3	6.67	7.41	6.36	1.05	0.69	1.21	0.95	0.00	0.00	0.00	0.00
	p4	5.71	6.06	5.00	0.00	0.00	1.74	0.00	1.00	4.44	20.00	NA
	p5	6.67	6.15	9.47	0.00	0.80	1.94	2.00	0.00	2.50	0.00	NA
	p6	4.44	5.71	5.16	0.95	0.00	0.00	0.00	6.67	1.67	0.00	NA
	**mean**	**5.54**	**6.16**	**5.82**	**0.33**	**0.46**	**1.16**	**0.69**	**1.72**	**1.67**	**6.67**	**0.00**
	**SD**	**1.18**	**0.76**	**1.97**	**0.52**	**0.53**	**0.68**	**0.83**	**2.64**	**1.67**	**10.33**	**0.00**
ES-2 (0.8 mV - 0.6 ms)	Pulse	Shank3			Shank2				Shank1			
		3,4*	***3,3****	***3,2****	***2.4***	***2.3***	***2.2***	***2.1***	***1.4***	***1.3***	***1.2***	***1.1***
	p1	2.22	3.38	2.67	0.00	1.74	1.54	2.35	1.05	1.67	10.00	NA
	p2	3.10	3.28	2.76	1.05	0.91	0.83	0.00	1.00	2.50	0.00	0.00
	p3	2.86	3.25	2.54	0.00	0.00	1.05	0.00	2.00	0.00	0.00	0.00
	p4	4.29	3.46	4.55	1.33	1.67	3.75	5.71	6.00	10.00	20.00	NA
	p5	7.06	7.27	7.44	1.67	0.00	0.00	2.22	0.00	0.00	0.00	NA
	p6	3.33	3.61	3.82	1.25	1.90	1.18	4.62	5.00	2.86	0.00	0.00
	**mean**	**3.81**	**4.04**	**3.96**	**0.88**	**1.04**	**1.39**	**2.48**	**2.51**	**2.84**	**5.00**	**0.00**
	**SD**	**1.73**	**1.59**	**1.88**	**0.71**	**0.87**	**1.26**	**2.34**	**2.42**	**3.71**	**8.37**	**0.00**
ES-3 (0.6 mV - 0.5 ms)	Pulse	Shank3			Shank2				Shank1			
		3,4*	***3,3****	***3,2****	***2.4***	***2.3***	***2.2***	***2.1***	***1.4***	***1.3***	***1.2***	***1.1***
	p1	9.09	8.33	6.67	3.53	2.11	1.54	0.00	0.00	0.00	0.00	NA
	p2	6.67	7.27	6.67	2.22	1.00	2.11	0.00	0.00	0.00	0.00	0.00
	p3	2.86	5.45	3.33	0.00	1.43	0.87	0.00	0.00	0.00	0.00	NA
	p4	23.33	23.33	16.00	1.43	1.43	1.25	2.50	0.00	0.00	0.00	NA
	p5	10.91	8.57	8.89	6.67	3.64	1.54	2.22	3.08	0.00	0.00	NA
	p6	8.75	7.50	6.67	1.18	0.00	2.50	2.86	0.00	0.00	0.00	NA
	**mean**	**10.27**	**10.08**	**8.04**	**2.50**	**1.60**	**1.63**	**1.26**	**0.51**	**0.00**	**0.00**	**NA**
	**SD**	**6.97**	**6.59**	**4.29**	**2.35**	**1.21**	**0.59**	**1.40**	**1.26**	**0.00**	**0.00**	**NA**
ES-4 (0.6 mV - 0.6 ms)	Pulse	Shank3			Shank2				Shank1			
		3,4*	***3,3****	***3,2****	***2.4****	***2.3***	***2.2***	***2.1***	***1.4***	***1.3***	***1.2***	***1.1***
	p1	3.11	4.29	3.41	0.00	1.05	1.54	0.00	0.00	0.00	NA	NA
	p2	4.33	5.26	5.77	1.33	0.00	2.50	0.00	0.00	0.00	NA	NA
	p3	2.22	2.89	2.19	0.00	0.00	0.00	0.00	2.22	0.00	0.00	NA
	p4	1.60	1.46	1.41	0.00	0.00	0.00	0.00	0.00	0.00	0.00	0.00
	p5	1.56	1.75	1.44	0.00	2.86	2.22	0.00	0.00	0.00	0.00	NA
	p6	1.22	1.59	1.36	0.00	0.00	1.11	0.00	0.00	0.00	0.00	NA
	**mean**	**2.34**	**2.87**	**2.60**	**0.22**	**0.65**	**1.23**	**0.00**	**0.37**	**0.00**	**0.00**	**NA**
	**SD**	**1.18**	**1.59**	**1.74**	**0.54**	**1.16**	**1.07**	**0.00**	**0.91**	**0.00**	**0.00**	**NA**
	Electrical stimulation efficiency (ESE)											
ES-5 (0.6 mV - 0.7 ms)	Pulse	Shank3			Shank2				Shank1			
		3,4*	***3,3****	***3,2***	***2.4***	***2.3***	***2.2***	***2.1***	***1.4***	***1.3***	***1.2***	***1.1***
	p1	0.70	0.92	0.44	0.00	3.08	0.00	4.00	0.00	0.00	0.00	0.00
	p2	5.66	5.71	4.39	5.00	2.86	2.00	3.33	0.00	0.00	0.00	NA
	p3	2.86	2.40	1.70	0.00	0.00	1.82	2.86	0.00	0.00	0.00	NA
	p4	5.79	5.95	4.71	3.33	4.29	8.00	0.00	0.00	0.00	0.00	0.00
	p5	10.67	10.71	11.43	1.43	0.00	1.54	0.00	1.18	1.43	5.00	0.00
	p6	4.06	3.78	4.13	1.18	2.86	1.00	2.86	1.54	0.00	20.00	0.00
	**mean**	**4.96**	**4.91**	**4.47**	**1.82**	**2.18**	**2.39**	**2.17**	**0.45**	**0.24**	**4.17**	**0.00**
	**SD**	**3.38**	**3.43**	**3.81**	**1.98**	**1.77**	**2.84**	**1.74**	**0.71**	**0.58**	**8.01**	**0.00**
ES-6 (0.6 mV - 0.8 ms)	Pulse	Shank3			Shank2				Shank1			
		3,4*	***3,3****	***3,2****	***2.4***	***2.3***	***2.2***	***2.1***	***1.4***	***1.3***	***1.2***	***1.1***
	p1	1.73	1.79	1.79	2.22	1.90	1.67	0.00	3.08	0.00	0.00	0.00
	p2	2.26	2.62	2.75	0.00	0.00	0.00	0.00	2.50	3.64	10.00	NA
	p3	2.86	2.62	2.81	2.67	1.67	4.29	4.00	2.00	3.33	0.00	0.00
	p4	2.20	2.12	2.15	1.00	1.67	1.33	4.00	0.00	0.00	0.00	0.00
	p5	1.40	1.27	1.36	0.00	1.18	1.00	0.00	1.25	3.33	5.00	NA
	p6	1.70	1.43	1.47	2.22	2.50	1.25	4.00	0.00	0.00	0.00	0.00
	**mean**	**2.02**	**1.98**	**2.05**	**1.35**	**1.49**	**1.59**	**2.00**	**1.47**	**1.72**	**2.50**	**0.00**
	**SD**	**0.52**	**0.58**	**0.63**	**1.19**	**0.85**	**1.44**	**2.19**	**1.29**	**1.88**	**4.18**	**0.00**

9 Supplementary Table 6

Supplementary Table 6. Significant stimulations in *rd10* retina. The ESEs of each recording electrode along the six electrical pulses for ES-2, ES-3, and ES-6 are displayed for the example shown in Figure 14. The average ESE and the corresponding standard deviation (SD) are also shown. Additionally, the electrodes that presented a significant stimulation (* = p<0.05) with a mean ESE >1 are marked in dark green, and those with a mean ESE <1 are marked with light green. "Inf" show the case when the firing rate after ES was increased but no activity was captured before ES, and "NA" stands for not applicable, indicating the cases where no activity was detected.

ES parameter	Electrical stimulation efficiency (ESE)															
ES-2 (0.8 mV - 0.6 ms)	Pulse	Shank4				Shank3				Shank2				Shank1		
		*4,4**	*4.3*	*4.2*	*4.1*	*3.4*	*3.3*	*3,2**	*3.1*	*2.4*	*2.3*	*2,2**	*2,1**	*1,4 **	*1,3**	*1,2**
	p1	4.00	1.08	1.05	1.36	5.00	2.58	2.26	1.36	0.00	1.43	1.30	1.43	2.06	2.36	2.38
	p2	3.08	1.48	1.77	1.51	0.00	3.81	2.82	1.66	6.67	6.67	3.23	2.90	2.29	2.77	2.82
	p3	1.43	1.54	0.78	0.48	0.00	1.00	0.51	0.76	0.00	0.87	1.76	1.10	2.08	1.85	2.11
	p4	5.71	1.60	0.96	0.92	13.33	5.45	2.22	1.30	0.00	2.86	1.48	1.68	2.40	2.69	2.76
	p5	2.22	0.67	1.57	2.24	6.67	0.74	2.04	1.35	20.00	1.82	2.46	2.83	1.82	2.34	2.84
	p6	1.82	1.57	0.96	0.82	20.00	1.11	2.05	1.26	20.00	3.81	2.90	3.03	2.52	2.85	3.54
	**mean**	**3.04**	**1.32**	**1.18**	**1.22**	**7.50**	**2.45**	**1.98**	**1.28**	**7.78**	**2.91**	**2.19**	**2.16**	**2.19**	**2.48**	**2.74**
	**SD**	**1.60**	**0.37**	**0.39**	**0.62**	**7.87**	**1.88**	**0.78**	**0.29**	**9.81**	**2.12**	**0.79**	**0.85**	**0.25**	**0.37**	**0.49**
ES-3 (0.6 mV - 0.5 ms)	Pulse	Shank4				Shank3				Shank2				Shank1		
		4,4	*4.3*	*4.2*	*4.1*	*3.4*	*3.3*	*3,2*	*3.1**	*2.4*	*2.3*	*2,2**	*2,1**	*1,4 **	*1,3**	*1,2**
	p1	0.00	1.00	0.71	0.79	0.00	0.00	1.00	0.71	0.00	1.11	0.93	1.64	1.72	1.98	2.14
	p2	2.00	0.41	0.85	0.85	0.00	0.00	0.89	0.78	0.00	0.95	2.03	1.15	0.92	1.01	1.33
	p3	0.00	1.05	2.11	1.41	0.00	1.36	0.74	1.03	0.00	0.63	1.98	1.93	1.71	1.84	2.02
	p4	0.00	1.00	1.23	1.03	0.00	2.22	1.72	0.78	10.00	2.22	2.31	2.56	1.54	1.68	1.90
	p5	1.54	0.00	0.44	0.56	0.00	0.00	0.35	0.71	0.00	0.77	1.69	1.26	1.14	1.24	0.90
	p6	0.00	0.39	0.51	0.55	20.00	0.59	0.51	0.65	0.00	2.67	1.71	1.71	1.42	1.68	1.54
	**mean**	**0.59**	**0.64**	**0.97**	**0.86**	**3.33**	**0.70**	**0.87**	**0.78**	**1.67**	**1.39**	**1.77**	**1.71**	**1.41**	**1.57**	**1.64**
	**SD**	**0.93**	**0.44**	**0.62**	**0.32**	**8.16**	**0.92**	**0.48**	**0.13**	**4.08**	**0.84**	**0.47**	**0.51**	**0.32**	**0.37**	**0.47**
ES-6 (0.6 mV - 0.8 ms)	Pulse	Shank4				Shank3				Shank2				Shank1		
		4,4	*4.3*	*4.2*	*4.1*	*3.4*	*3.3*	*3,2**	*3.1*	*2.4*	*2.3**	*2,2**	*2,1**	*1,4 **	*1,3**	*1,2**
	**p1**	0.00	0.92	0.86	1.11	0.00	2.31	2.37	1.68	5.00	6.00	2.18	2.29	1.85	2.11	2.03
	**p2**	4.00	1.14	1.50	1.12	0.00	2.07	1.10	1.96	10.00	0.95	2.05	1.40	1.87	1.91	2.11
	**p3**	1.43	2.26	1.22	0.87	0.00	0.67	1.27	3.27	0.00	2.00	1.39	1.27	2.02	2.09	2.12
	**p4**	2.86	1.05	1.49	1.51	NA	3.48	1.47	1.60	0.00	4.00	2.06	3.26	2.76	2.61	2.87
	**p5**	0.00	0.43	0.94	1.04	Inf	3.33	2.73	0.90	0.00	5.00	2.90	1.34	2.52	2.68	2.93
	**p6**	0.00	0.67	0.75	1.04	0.00	0.00	2.22	1.26	NA	2.50	3.16	2.53	2.08	2.38	2.73
	**mean**	**1.38**	**1.08**	**1.13**	**1.11**	**0.00**	**1.98**	**1.86**	**1.78**	**3.00**	**3.41**	**2.29**	**2.02**	**2.18**	**2.29**	**2.46**
	**SD**	**1.72**	**0.63**	**0.32**	**0.21**	**0.00**	**1.40**	**0.67**	**0.82**	**4.47**	**1.92**	**0.64**	**0.81**	**0.37**	**0.31**	**0.42**

10 Supplementary Figure 4

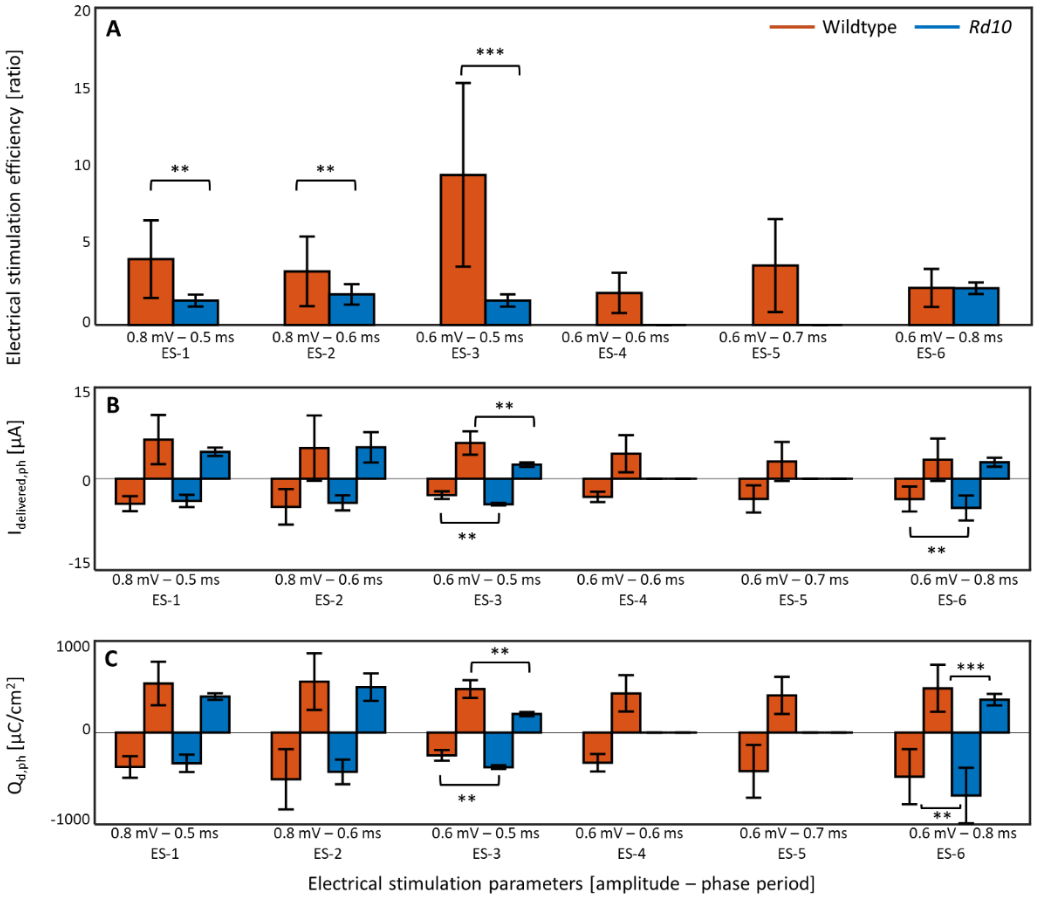

Supplementary Figure 4. Electrical stimulation in wildtype and *rd10* retinas. In (A), the electrical stimulation efficiency ratio of the stimulated shank (average of recording electrodes) for six different stimulation parameters (ES-1 to ES-6) tested successfully on two different wildtype retinas and ES-3 generated a significant response only on one retina. Likewise, ES-2 evoked successful electrical responses on two *rd10* retinas, and ES-1, ES-3, and ES-6 each one on one retina. The average cathodic and anodic delivered current I_{del} (B) and charge density $Q_{d,ph}$ (C) is shown for both wildtype (in orange) and *rd10* retinas (in blue) during the ES parameters that lead to successful stimulations with an increased firing rate. Significant ESE differences between wildtype and *rd10* retinas are shown for ES-1, ES-2, and ES-3. Likewise, significant I_{del} and Q_d differences are shown for ES-3 and ES-6 (** = $p<0.01$, *** = $p<0.001$, Wilcoxon rank sum test).

11 Supplementary Table 7

Supplementary Table 7. Electrical stimulation thresholds. Upper and lower threshold values of the average cathodic and anodic delivered current ($I_{del,c/a}$) and charge density ($Q_{d,c/a}$) that lead to significant electrical stimulations with an increased firing rate for wildtype and *rd10* retinas.

Retina Type	Threshold	$I_{del,c}$ [µA]	$I_{del,a}$ [µA]	$Q_{d,c}$ [µC/cm^2]	$Q_{d,a}$ [µC/cm^2]
**Wildtype**	Lower	-2.69 ± 0.62	2.84 ± 3.21	-248.28 ± 58.96	406.21 ± 202.78
	Upper	-4.62 ± 2.92	6.43 ± 4.04	-508.55 ± 329.14	555.69 ± 308.41
Rd10	Lower	-3.64 ± 1	2.32 ± 2.5	-334.76 ± 94.65	202.69 ± 23.2
	Upper	-4.79 ± 2.06	5.17 ± 0.39	-686.10 ± 304.16	495.59 ± 150. 37
